# Elucidating the Metabolism of Chiral PCB95 in Wildtype and Transgenic Mouse Models with Altered Cytochrome P450 Enzymes Using Intestinal Content Screening

**DOI:** 10.1021/acs.chemrestox.4c00350

**Published:** 2024-11-19

**Authors:** Xueshu Li, Amanda J. Bullert, Binita Gautam, Weiguo Han, Weizhu Yang, Qing-Yu Zhang, Xinxin Ding, Hans-Joachim Lehmler

**Affiliations:** † Department of Occupational and Environmental Health, College of Public Health, University of Iowa, Iowa City, Iowa 52242, United States; ‡ Interdisciplinary Graduate Program in Neuroscience, University of Iowa, Iowa City, Iowa 52242, United States; § Department of Pharmacology and Toxicology, College of Pharmacy, University of Arizona, Tucson, Arizona 85721, United States

## Abstract

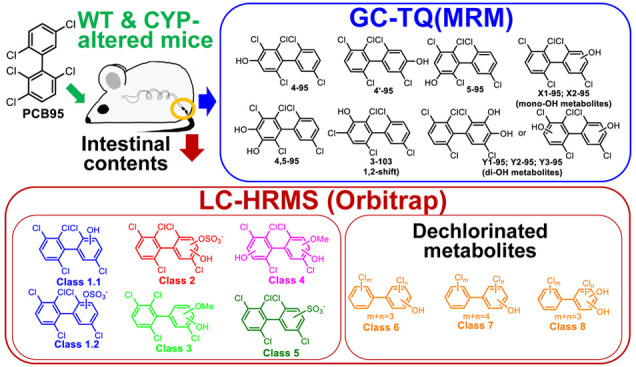

Polychlorinated biphenyls (PCBs), such as 2,2′,3,5′,6-pentachlorobiphenyl (PCB95), are persistent organic pollutants associated with adverse health outcomes, including developmental neurotoxicity. PCB95 is a chiral neurotoxic PCB congener atropselectively metabolized to potentially neurotoxic metabolites in vivo. However, the metabolic pathways of most PCB congeners, including PCB95, remain unknown. To address this knowledge gap, we analyzed the intestinal contents of mice exposed to PCB95 to elucidate the PCB95 metabolism pathway and assess if genetic manipulation of hepatic drug-metabolizing enzymes affects PCB95 metabolism. Our study exposed male and female wildtype (WT), *Cyp2abfgs*-null (KO), and CYP2A6-transgenic/*Cyp2abfgs-null* (KI) mice orally to 1.0 mg/kg body weight of PCB95. Intestinal content was collected 24 h after PCB administration. aS-PCB95 was enriched in all intestinal content samples, irrespective of sex and genotype. Gas chromatography–tandem mass spectrometry (GC–MS/MS) analyses identified 5 mono- (OH-PCB95) and 4 dihydroxylated PCB (diOH-PCB95) metabolites. Liquid chromatography–high-resolution mass spectrometry (LC–HRMS) identified 15 polar hydroxylated, methoxylated, and sulfated PCB95 metabolites, including 3 dechlorinated metabolites. A sex difference in the relative OH-PCB95 levels was observed only for KO in the LC–HRMS analysis. Genotype-dependent differences were observed for female, but not male, mice, with OH-PCB95 levels in female KO (F_KO_) mice tending to be lower than those in female WT (F_WT_) and KI (F_KI_) mice. Based on the GC–MS/MS analysis, these differences are due to the unknown PCB95 metabolites, X1-95 and Y1-95. These findings demonstrate that combining GC–MS/MS analyses and LC–HRMS subject screening of the intestinal content of PCB95-exposed mice can significantly advance our understanding of PCB95 metabolism in vivo.

## Introduction

Polychlorinated biphenyls (PCBs) are manmade persistent organic pollutants^1^ that were widely used in various applications, such as hydraulic fluids and electric equipment.^2^ Although the production of PCBs has been banned due to their toxic and persistent nature, some PCB congeners continue to be produced inadvertently,^3^ resulting in ongoing environmental contamination and human exposure. PCBs persist in the environment, leading to widespread contamination of air, water, sediments, and soil.^4^ They bioaccumulate and biomagnify in aquatic and terrestrial food chains, posing health risks to humans and wildlife. The general population is exposed to PCBs via the diet or by inhalation of PCB-contaminated indoor and outdoor air.^5^ Exposure to PCBs is associated with several adverse health effects, for example, developmental abnormalities,^6^ neurotoxicity,^7−9^ immune system impairment,^9,10^ and reproductive disorders.^11,12^ The International Agency for Research on Cancer (IARC) classifies PCBs as human carcinogens.^13^

PCB95 is a major component of technical PCB mixtures and has been implicated in PCB developmental neurotoxicity. It is present in post-mortem human brain tissue^14^ and in the serum of children and their mothers.^15^ It is a chiral PCB congener that displays axial chirality because of the hindered rotation around the phenyl–phenyl bond due to the presence of three *ortho* chlorine substituents.^16^ PCB95 is atropselectively oxidized to hydroxylated metabolites by CYP2 family enzymes, such as human CYP2A6,^17−19^ resulting in nonracemic PCB95 and OH-PCB95 residues, for example, in the earthworm *Eisenia fetida*,^20^ mice,^21^ and rats.^22^ PCB95 is a potent sensitizer of ryanodine receptors^23^ and promotes dendritic growth.^24,25^ Pure PCB95 atropisomers display atropselectivity toward ryanodine receptors and atropselectively influence hippocampal neuronal networks.^26^

OH-PCBs are conjugated by phase II enzymes to more polar PCB metabolites that are excreted in urine^27,28^ and feces.^28,29^ For example, OH-PCBs can be conjugated to sulfate metabolites and glucuronides by sulfotransferases (SULT) and UDP-glucuronosyltransferases (UGT), respectively.^30,31^ PCB sulfates are present in laboratory animals and humans and display toxicities that differ from those of the parent PCBs.^32^ These PCB metabolites can be further oxidized by cytochrome P450 enzymes, resulting in hydroxylated OH-PCBs, PCB sulfates, and PCB glucuronides. Dihydroxylated PCB metabolites can be methylated by catechol-*O*-methyltransferase (COMT), resulting in methoxylated PCB metabolites.^29,33,34^ Alternatively, dihydroxylated PCBs undergo sulfation and glucuronidation reactions, resulting in metabolites that may undergo further oxidation reactions. Unfortunately, the metabolic pathways of many PCB congeners, such as PCB95, in wildlife, laboratory animals, and humans remain unknown. Because the complex PCB metabolite mixtures formed in vitro or in vivo cause cellular dysfunction, as we have shown in HepG2 cells in culture,^31^ there is a need to elucidate the metabolic pathways of individual PCB congeners in vivo and explore the role of specific drug-metabolizing enzymes in PCB metabolism and toxicity.

Liquid chromatography–high-resolution mass spectrometry (LC–HRMS) has been used to characterize the complex PCB metabolite mixture in biological samples, such as polar bear serum.^35^ However, the analysis of PCB metabolites in serum is not ideal for characterizing PCB metabolic pathways because some PCB metabolites have a short serum half-life, as shown for PCB11 sulfate following intravenous administration^36^ or are not transported into target tissues. Because PCB metabolites with more than two chlorine substituents are primarily eliminated with the feces,^37^ we characterized the PCB95 metabolites in intestinal content from mice exposed orally to PCB95. We used wildtype, *Cyp2abfgs*-null mice (mice with a broad deletion of multiple *Cyp2* genes involved in the metabolism and toxicity of endogenous and exogenous compounds),^38^ and CYP2A6-humanized mice (mice with human CYP2A6 but no mouse *Cyp2abfgs* gene)^39^ to determine if PCB metabolite profiles in the gastrointestinal tract are affected by the presence or absence of specific CYP enzymes, such as human CYP2A6, a cytochrome P450 enzyme involved in the metabolism of PCB95.^17−19^ This approach allowed us to characterize the complex metabolic pathway of PCB95 in mice for the first time and gain insights into the role of specific cytochrome P450 enzymes in PCB95 metabolism in vivo.

## Experimental Section

### Chemicals and Materials

Racemic PCB95 was prepared by the Suzuki coupling reaction of 1,2,4-trichloro-3-iodobenzene and 2,5-dichlorobenzeneboronic acid.^40^ 3-Methoxy-2,2′,4,5′,6-pentachlorobiphenyl (methylated derivative of 3-103), 2,2′,3,5′,6-pentachlorobiphenyl-5-ol (5-95), 2,2′,3,5′,6-pentachlorobiphenyl-4′-ol (4′-95), 2,2′,3,5′,6-pentachlorobiphenyl-4-ol (4-95), 4,5-dimethoxy-2,2′,3,5′,6-pentachlorobiphenyl (methylated derivative of 4,5-95), and sulfuric acid mono-(2,2′,5,5′-tetrachlorobiphenyl-4-yl) ester, ammonium salt (4-PCB52 sulfate) were synthesized as described previously.^41,42^ 2,3,4′,5,6-Pentachlorobiphenyl (PCB117), 2,2′,3,4,4′,5,6,6′-octachlorobiphenyl (PCB204) and 2,3,3′,4,5,5′-hexachlorobiphenyl-4′-ol (4′-159) were purchased from AccuStandard, Inc. (New Haven, CT, USA). Polar standards for LC–HRMS analysis, including 4′-chloro-3′-fluorobiphenyl-4-ol (3-F,4′-OH-PCB3) and sulfuric acid mono-(4′-chloro-3′-fluorobiphenyl-4-yl) ester, ammonium salt (3-F,4′-PCB3 sulfate), were provided by the Synthesis Core of Iowa Superfund Research Program.^27^ Perfluorooctanesulfonic acid (PFOS) potassium salt was purchased from Thermo Fisher Scientific (Pittsburgh, Pennsylvania, USA). Diazomethane in diethyl ether was synthesized by the Synthesis Core of the Iowa Superfund Research Program.^43^ The abbreviations of the PCB metabolites are an abbreviated version of the PCB metabolite nomenclature proposed by Maervoet and co-workers.^44^ The unique identifiers of PCBs and metabolites are summarized in Table S1. All data underlying this manuscript are available free of charge on Iowa Research Online at https://doi.org/10.25820/data.00744.^45^

### Generation and Maintenance of CYP2A6-Humanized Mouse Models

The animal experiments were performed following the Guide for the Care and Use of Laboratory Animals of the National Research Council and were performed at the University of Arizona (Tucson, AZ, USA). The studies were approved by the Institutional Animal Care and Use Committees of the University of Arizona under protocol #17-355. *Cyp2abfgs*-null (KO mice)^46^ and CYP2A6-transgenic mice/*Cyp2abfgs*-null (KI mice)^47^ on a C57BL/6 genetic background and congenic wildtype (WT) mice were obtained from breeding colonies maintained in Association for Assessment and Accreditation of Laboratory Animal Care (AALAC)-approved facilities at the University of Arizona. *Cyp2abfgs*^–/–^ mice and CYP2A6^+^ mice were intercrossed to yield CYP2A6^+^/*Cyp2abfgs* mice. Intercrossing of these CYP2A6^+^/*Cyp2abfgs*^+/–^ mice with *Cyp2abfgs*^–/–^ mice generated *Cyp2abfgs*^–/–^ null mice (KO mice) and CYP2A6^+^/*Cyp2abfgs*^–/–^ humanized littermates (KI mice). Genotype analysis for the human CYP2A6 transgene and the mouse *Cyp2abfgs* genes were performed following published protocols.^46,47^ The mice were given access to food and water ad libitum and were kept in a controlled environment with a 12 h light/dark cycle and regulated airflow, temperature, and light. No adverse events were observed throughout the study.

### Animal Exposure

The intestinal content samples used in this study were generated as part of a published study of the atropselective disposition of PCB95 and its hydroxylated metabolites in mice.^48^ Briefly, 4 month-old adult male and female WT, KO, and KI mice (M_WT_, *n* = 6; M_KO_, *n* = 7; M_KI_, *n* = 7; F_WT_, *n* = 7; F_KO_, *n* = 7; F_KI_, *n* = 5) were exposed by oral gavage to a single dose of racemic PCB95 (1.0 mg/kg bw) in stripped corn oil (10 mL/kg; lot# A0395699; cat# 801-03-7; Fisher Scientific) as the vehicle. This PCB dose is frequently used in PCB developmental neurotoxicity studies in rodent models.^21,49−52^ Blank tissue samples to assess the background contamination with PCB95 were generated in parallel by administering corn oil alone to mice (1–3 mice per group). The animals were euthanized 24 h after exposure, and intestinal content samples from the dissected distal colon and rectum were collected. Samples were stored at −80 °C in separate plastic bags and shipped on dry ice to the University of Iowa for analysis.

### Extraction of PCB95 and Its Total Hydroxylated Metabolites from Intestinal Content

PCB95 and total OH-PCB95 metabolite levels were determined in intestinal content samples after deconjugation of sulfate and glucuronide conjugates using published methods with modifications.^53^ Briefly, intestinal content samples (50 ± 22 mg, *n* = 38) were homogenized with a TissueRuptor (Qiagen, Hilden, Germany) in 3 mL of 0.2 M sodium acetate buffer (pH 5) in glass tubes and spiked with 4-PCB52 sulfate (100 ng in DMSO) as a surrogate standard. After adding 50 μL of sulfatase (type H-2 from *Helix pomatia*, Sigma-Aldrich, Burlington, MA, USA), the samples were kept at 37 °C for 16 h in a shaking water bath. Samples were quenched with 1 mL of 6 M HCl, and the surrogate standards (50 ng of PCB117 in isooctane and 50 ng of 4′-159 in methanol) were added to the incubation system. Subsequently, 5 mL of 2-propanol and 5 mL of a 1:1 hexane–MTBE mixture (v/v) were added. The samples were inverted for 5 min and centrifuged at 1690*g* for 5 min to facilitate the phase separation. Next, the organic phases were transferred to new glass tubes with 3 mL of 1% KCl solution, the aqueous phases were re-extracted with 3 mL of hexane, and the organic phases were combined with the above extract. After inverting and centrifugation, the organic phases were collected, and the aqueous KCl phases were re-extracted with 3 mL of hexane. The combined extracts were evaporated to near dryness using a Savant SpeedVac SPD210 vacuum concentrator (Thermo Scientific, Waltham, MA, USA) coupled with an RVT5105 refrigerated vapor trap (Thermo Scientific, Waltham, MA, USA), and each sample was reconstituted with 0.5 mL of hexane.

### Derivatization of OH-PCBs with Diazomethane and Sample Cleanup

Five drops of methanol and 0.5 mL of diazomethane (about 5 mmol) in diethyl ether were added to the intestinal content extracts to derivatize the OH-PCBs.^43^ The samples were stored at 4 °C for approximately 16 h for derivatization, and excess diazomethane was removed under a gentle stream of nitrogen in a fume hood. The extract was reconstituted to 0.5 mL of dichloromethane and was loaded onto a glass SPE cartridge filled with 2 g of acidified silica gel (silica gel/H_2_SO_4_, 2:1, w/w) with 0.2 g of deactivated silica gel on the bottom.^14^ The PCB and metabolites were eluted from the SPE cartridge with 14 mL of dichloromethane; the eluent was concentrated with a Savant SpeedVac SPD210 vacuum concentrator (Thermo Scientific) to near dryness, and the solvent was exchanged to 4 mL of hexane. The extracts were treated with 4 mL of concentrated sulfuric acid for further lipid removal.^14^ The organic phase was concentrated to near dryness with a Savant SpeedVac SPD210 vacuum concentrator (Thermo Scientific) and spiked with an internal standard (50 ng of PCB204 in isooctane) for gas chromatographic quantification of PCB95 and its hydroxylated metabolites (as methylated derivatives).

### Gas Chromatographic Determinations

PCB95 and metabolite determinations were conducted on an Agilent 7890B gas chromatograph equipped with an Agilent 7000D Triple Quad and an Agilent 7693 autosampler in the multiple reaction monitoring setting (GC–MS/MS).^14^ Gas chromatographic separations were performed with an SPB-Octyl capillary column (30 m length, 25 mm inner diameter, 0.25 μm film thickness; Supelco, Bellefonte, PA, USA). Samples were injected in the solvent vent mode with a helium (carrier gas) flow of 0.8 mL/min. Nitrogen was used as the collision gas. The following temperature program^14^ was used for the separation of PCB95 and its metabolites: Initial temperature of 45 °C, held for 2 min, 100 °C/min to 75 °C, held for 5 min, 15 °C/min to 150 °C, held for 1 min, 2.5 °C/min to 280 °C, and final hold of 5 min. The transfer line temperature was 280 °C. The multiple reaction monitoring (MRM) method settings, including mass ion transitions, dwell time, and collision energy, are summarized in Table S2. Analytes were identified based on the MRM transition and the retention time compared to the authentic standard. Levels were determined using the internal standard method and adjusted for the recovery of the respective surrogate standard.^54^ Unknown metabolites were tentatively identified based on the MRM transition. The average relative response factor for the available hydroxylated PCB (OH-PCB) metabolite standards (as methylated derivatives) was used to estimate the levels of the unknown PCB95 metabolites.^14^

Atropselective analyses of PCB95 were performed using an Agilent 7890A gas chromatograph with a ^63^Ni-μECD detector and a Chirasil-Dex CB capillary column (25 m length, 250 μm inner diameter, 0.25 μm film thickness; Agilent, Santa Clara, CA, USA).^52^ The following temperature program was used: 50 °C, hold for 1 min, 10 °C/min to 150 °C, hold for 65 min, 15 °C/min to 200 °C, and hold for 15 min. The injector and detector temperatures were 250 °C. Helium was used as carrier gas with a flow rate of 3.0 mL/min; argon-methane 5% was used as makeup gas with a 60 mL/min flow rate. The enantiomeric fraction (EF) values were calculated with the valley drop method^55^ as EF = *A*_E1_/(*A*_E1_ + *A*_E2_), where *A*_E1_ and *A*_E2_ are the peak area of the first (E1) and the second eluting (E2) atropisomers, respectively.

### Semitargeted Analysis of PCB95 Metabolites in Intestinal Content

A subset of intestinal content samples (18–33 mg, *N* = 30) were selected for semitargeted LC–HRMS.^14^ Aliquots were homogenized in a glass tube with 2 mL of Milli-Q water using a TissueRuptor. The homogenates were spiked with a mixture of surrogate standards (3-F,4′-OH-PCB3 and 3-F,4′-PCB3 sulfate, 50 ng each in acetonitrile). 4 mL of acetonitrile with 1% formic acid was added, and the homogenates were vortexed for 10 s. 200 mg of sodium chloride and 800 mg of magnesium sulfate were added, and the samples were shaken vigorously, inverted for 5 min, and centrifuged at 1181*g* for 5 min to facilitate phase separation. The organic phase on the top was passed through a 3 mL HybridSPE cartridge (Supelco, Inc., Bellefonte, PA, USA) loaded with 3 g of a mixture of anhydrous sodium sulfate and anhydrous magnesium sulfate (1:1, w/w) and preconditioned with 3 mL of acetonitrile. The aqueous phase was re-extracted with an additional 1 mL of acetonitrile, and the organic phase was also loaded onto the Hybrid SPE cartridge. The PCB metabolites were eluted with 3 mL of acetonitrile. The combined eluents were evaporated to dryness using a Savant SpeedVac SPD210 vacuum concentrator (Thermo Scientific) at 35 °C. The residual extracts were redissolved in 300 μL of acetonitrile and transferred to microcentrifuge tubes. Potassium perfluorooctanesulfonate (PFOS, 100 ng in acetonitrile) was spiked onto the samples as a volume corrector. The solvent was evaporated to dryness using a Savant SpeedVac SPD210 vacuum concentrator (Thermo Scientific), and the extracts were reconstituted with 200 μL of solvent (water–acetonitrile = 50–50, v %) and kept in a −20 °C freezer for 30 min to facilitate precipitation. Then, the extracts were vortexed for 10 s and centrifuged for 10 min at 4 °C and 16,000*g* to precipitate the protein. Then, the supernatants were transferred to autosampler vials with an insert and kept at −80 °C until LC–HRMS analysis.

Samples were analyzed on a Q-Exactive Orbitrap mass spectrometer (Thermo Fisher Scientific, Waltham, MA, USA) with a Vanquish Flex ultrahigh-performance liquid chromatograph (Thermo Fisher Scientific) with an ACQUITY UPLC-C18 column (particle size: 1.7 μm, inner diameter 2.1 mm, length 100 mm, Waters, Milford, MA, USA). Water (10 mM ammonium format and 0.1% formic acid) and acetonitrile (10 mM ammonium format and 0.1% formic acid) were used as mobile phases A and B, respectively. The mobile flow rate was 0.3 mL/min, and the pressure was 4000–8500 psi with a column chamber temperature of 25 °C. The UPLC gradient program was as follows: starting at 5% B, held for 1 min, then increased linearly to 95% B, held for 3 min, and returning to 5% B, with a hold for 4 min before the next injection.^14^ The injection volume was 2 μL. The LC–HRMS instrument was operated in the negative polarity mode. The analyses were performed in the full scan mode (range: 150–1000 *m*/*z*) with the following source parameter settings: spray voltage, 2472 V; spray current, 18.2 μA; capillary temperature, 256 °C; sheath gas flow rate, 48; auxiliary gas flow rate, 2; and auxiliary temperature, 413 °C. The Automatic Gain Control target and the full scan resolution settings were 1 × 10^6^ and 70, 000 respectively, and the maximum interval time was 200 ms. PCB95 metabolites were putatively identified based on a subject screening list based on earlier PCB metabolism studies,^27,29,30,35^ with a 5 ppm mass tolerance and 5 mass precision digitals on Xcalibur 4.1 (Thermo Fisher Scientific Inc., Waltham, MA, USA). The identification was further confirmed using the chlorine isotope patterns of the molecular ion [M-H]^−^ and, if applicable, characteristic ion fragments. Putative PCB95 metabolites are reported if they were detected in more than 50% of the samples.

### Quality Assurance and Quality Control (QA/QC)

Method blanks and blank tissue samples were extracted in parallel with each sample batch for GC–MS/MS and LC–HRMS analysis. For the GC–MS/MS analyses, the method detection limits (MDL) were determined based on the method blanks (Table S3). Limits of quantification (LOQ) in different matrices were established based on the corresponding tissue blanks (Table S3). PCB sulfate surrogate standard, 4-PCB52 sulfate (measured after deconjugation as the methylated derivative of 4-95), PCB surrogate standard (PCB117), and OH-PCB surrogate standard (4′-159) were added to each sample to assess the precision and reproducibility of the GC–MS/MS analysis (Table S4). In addition, an ongoing precision and surrogate standard was extracted in parallel from both the method and tissue blanks with each sample batch (Table S5). The EF value of the racemic standard of PCB95 was 0.498 ± 0.004 (*n* = 7). For LC–HRMS analysis, 3-F,4′-OH-PCB3, and 3-F,4′-PCB3 sulfate were used as surrogate standards. The recoveries of these standards in method blanks and extracts from the intestinal content are summarized in Table S6.

### Data Analysis

Tissue levels, determined by GC–MS/MS, and EF values are reported as mean ± standard deviation (Table S7 and Figure S1). The relative abundance of metabolites detected by LC–HRMS is also presented as the mean ± standard deviation (Table S8). The statistical analyses used individual mice as the statistical unit and were performed with two-way ANOVA with the Bonferroni model in GraphPad Prism 9.4.1 (Tables S9 and S10). In addition, OH-PCB metabolite profiles were compared using the similarity coefficient, cos θ (Figure S2).^56^ The cos θ ranges from 0 to 1, where a value of 0 indicates completely different profiles and a value of 1 indicates identical profiles.

## Results and Discussion

### PCB95 Levels in the Intestinal Content

**Figure 1:**
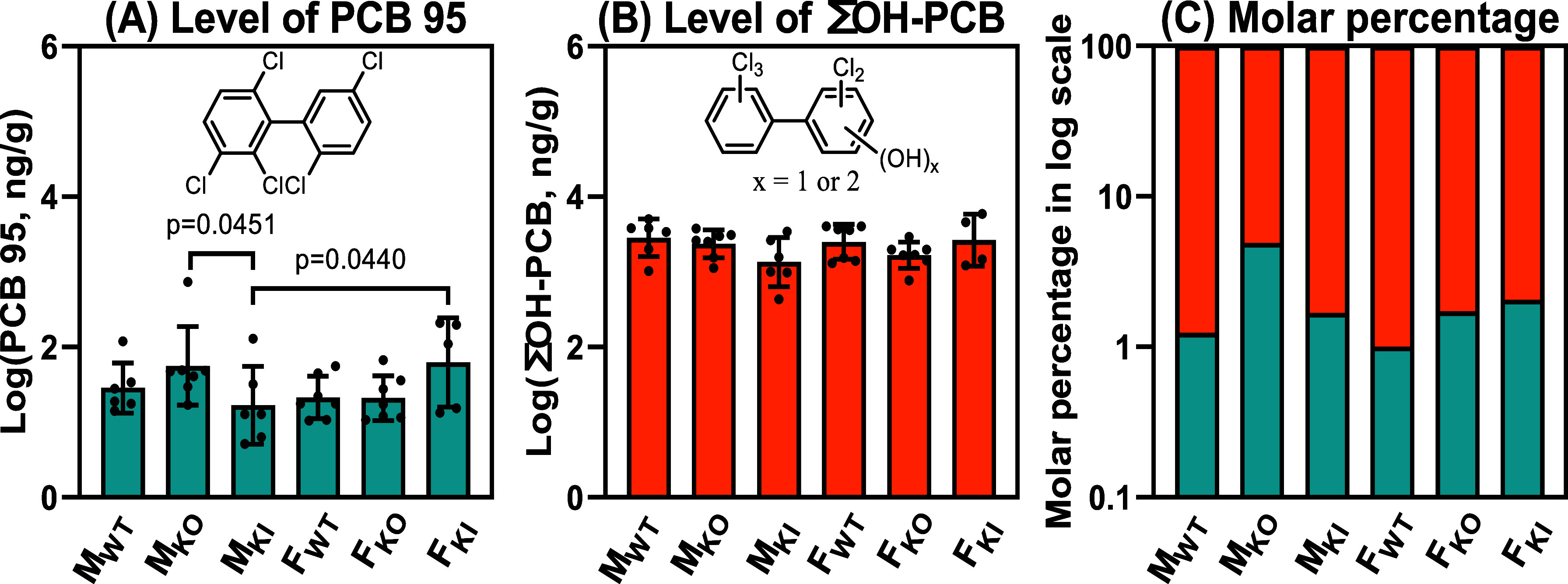
Comparison of the levels of (A) PCB95, (B) ΣOH-PCB95 (ng/g), and (C) molar percentage of PCB and ΣOH-PCBs in the intestinal content from male and female wildtype, *Cyp2abfgs*-null (KO), and CYP2A6-humanized (KI) mice exposed to PCB95 reveals sex and genotype-dependent differences in the disposition of PCB95 (*n* = 5–7 per exposure group). Levels of PCB95 and ΣOH-PCBs are averages ±standard deviation and presented on a logarithmic scale. Statistical analyses were performed by two-way ANOVA with the Bonferroni correction for multiple comparisons, with *p* < 0.05 considered significant. M_WT_, male wildtype; M_KO_, male *Cyp2abfgs*-null; M_KI_, male CYP2A6-humanized; F_WT_, female wildtype; F_KO_, female *Cyp2abfgs*-null; F_KI_, female CYP2A6-humanized mice.

Feces is a minor route of elimination of the parent PCB95 and related congeners in laboratory animal models.^57−59^ In the present study, PCB95 was detected in all intestinal content samples, with levels ranging from 26 to 139 ng/g of wet weight in the different exposure groups (Figure 1A). The PCB95 detected in the intestinal content represents PCB95 that was not absorbed, excreted via the bile, or passively diffused from the systemic circulation into the gastrointestinal tract.

We observed some genotype and sex differences in the PCB95 levels. PCB95 levels in the intestinal content were significantly higher in M_KO_ vs M_KI_ mice. Levels were also higher in M_KO_ mice than in M_WT_ mice; however, this difference was not statistically significant. The higher PCB95 content in the intestinal content of M_KO_ mice compared to male mice from the other two genotypes is consistent with reduced metabolism of PCB95 due to the deletion of the *Cyp2abfgs* genes. The lower PCB95 levels in the intestinal content of M_KI_ mice than those in M_KO_ mice are consistent with the role of CYP2A6 in the oxidation of PCB95. Moreover, F_KI_ mice had PCB95 levels higher than those of M_KI_ mice. These differences may be due to genotype or sex differences in the metabolism or excretion of PCB95. Alternatively, differences in physiological parameters, such as the gastrointestinal transit time, may explain the differences in the PCB95 levels between M_KI_ vs F_KI_ mice.

### Enantiomeric Fractions of PCB95 in the Intestinal Content

PCB95 displays axial chirality due to the hindered rotation around the phenyl–phenyl bond in the presence of three *ortho* chlorine substituents.^60^ Changes in the atropisomeric enrichment of PCBs can provide powerful insights into biological processes in the environment or in vivo.^61^ In the present study, the PCB95 atropisomer eluting second on the chiral column was enriched in all intestinal content samples analyzed. This PCB atropisomer corresponds to aS-PCB95.^19^ The EF values of PCB95 ranged from 0.39 in F_WT_ mice to 0.45 in M_KO_ mice and did not significantly differ by genotype or sex (Figure S1). An enrichment of aS-PCB95 was also observed in tissues from pregnant and nonpregnant mice exposed for 56 or 39 days orally to different doses of PCB95.^21,52^ The enantiomeric enrichment of aS-PCB95 indicates that some of the PCB detected in the intestinal content underwent enantioselective metabolism before being excreted, most likely by passive diffusion, into the gastrointestinal tract.^37,62^ Moreover, because mice have a gastrointestinal transit time of approximately 14 h,^63^ some of the PCB95 detected in intestinal content 24 h after PCB exposure is due to unresorbed compounds, as discussed above.

The enantiomeric enrichment of PCB95 in mammals is due to the atropselective oxidation of PCB95 by cytochrome P450 enzymes. To date, the direction of the enantiomeric enrichment of PCB95 by mouse cytochrome P450 enzymes has not been reported in vitro. However, aR-PCB95 is preferentially metabolized by rat CYP2B1 enzymes, resulting in an enrichment of aS-PCB95 analogous to the enrichment observed in the present study.^64^ In contrast, metabolism studies with human liver microsomes and recombinant human cytochrome P450 enzymes demonstrate enrichment of aR-PCB95.^17−19^ Based on these in vitro studies, KI mice are predicted to display enrichment of aR-PCB95 in the intestinal content; however, this is not the case (Figure S1). This apparent discrepancy may be due to the atropselective biotransformation of PCB95 by cytochrome P450 enzymes other than CYP2 enzymes in male and female KI mice. Our earlier in vitro metabolism and in vivo disposition studies support this hypothesis. For example, our microsomal metabolism studies revealed that CYP2F2, along with other unidentified hepatic cytochrome P450 enzymes, metabolizes PCB95 and structurally related PCB congeners, resulting in the enrichment of one atropisomer.^65^

### Total OH-PCB95 Levels in the Intestinal Content

OH-PCB95 levels were measured by GC–MS/MS to gain initial insights into the oxidative metabolism of PCB95 in the mouse strains used in this study.^66^ The sum of OH-PCB95 metabolites (ΣOH-PCBs) in intestinal content was 40 to 110-fold higher than the levels of the parent compound, ranging from 1600 to 4900 ng/g wet weight (Figure 1C; Table S7). The ΣOH-PCB levels in the intestinal content in M_KO_ mice were one exception and were only 18-fold higher than the PCB95 levels. On a molar basis, the percentage of ΣOH-PCB relative to PCB95 was also clearly different in M_KO_ mice (Figure 1C). Genotype or sex did not significantly affect the ΣOH-PCB levels, possibly because of the large variability in the OH-PCB levels. These results are consistent with earlier reports that feces is a major route of the excretion of the metabolites of PCB95 and structurally related PCB congeners in rodents.^53,58,67^

### Detection of Individual OH-PCB95 Congeners in the Intestinal Content

**Figure 2:**
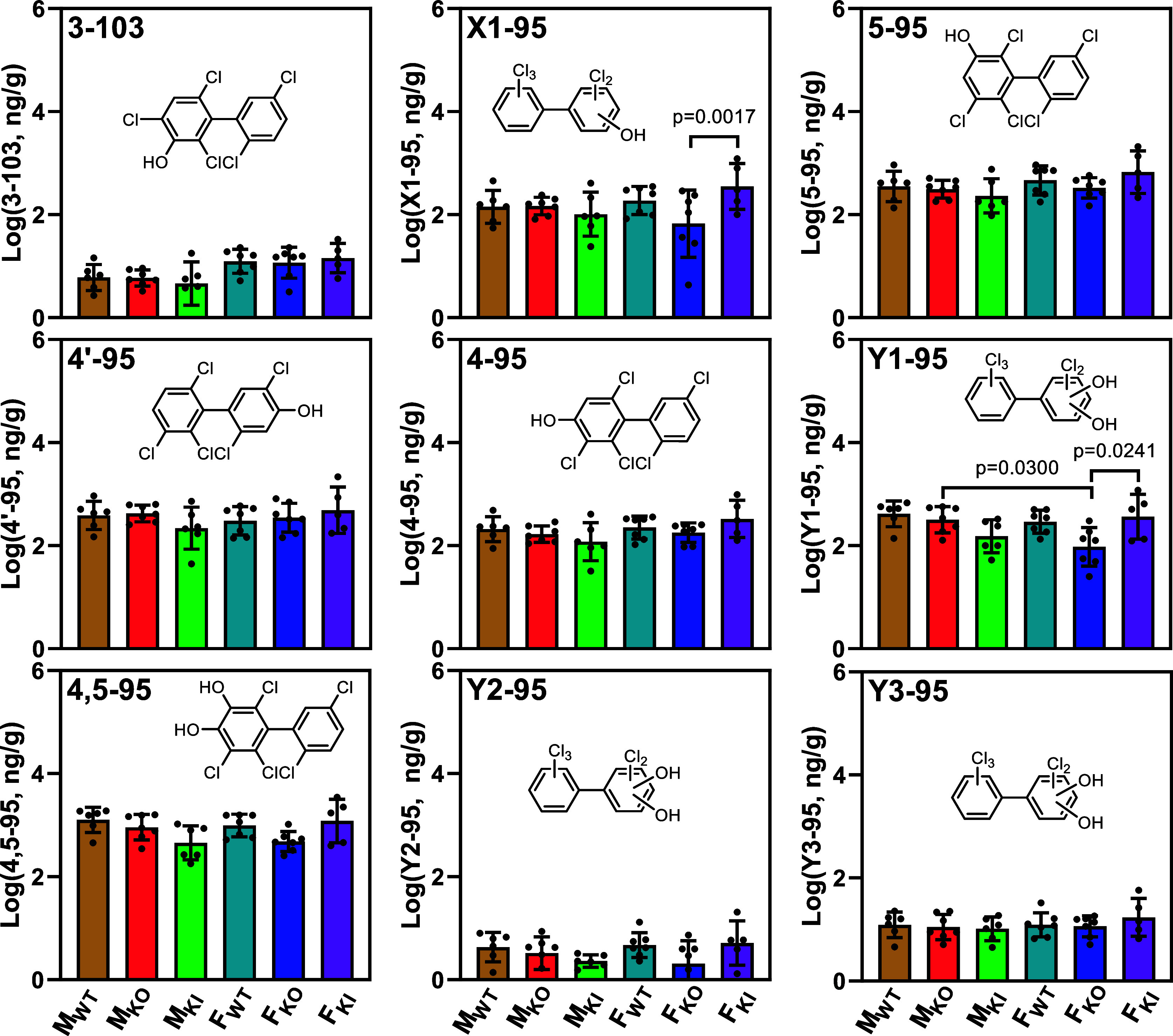
Levels (ng/g) of mono- and dihydroxylated PCB95 metabolites in the intestinal content from male and female wildtype (WT), *Cyp2abfgs*-null (KO), and CYP2A6-humanized (KI) mice exposed to PCB95 reveal some sex and genotype-dependent differences in the disposition of PCB95 (*n* = 5–7 per exposure group). X1-95 is an unknown monohydroxylated PCB95 metabolite; Y1-95, Y2-95, and Y3-95 are unknown dihydroxylated PCB95. The unknown PCB95 metabolites are tentatively identified based on their MRM transition and quantified using the average relative response factors (RRF) of the available standards with the same transition (i.e., the average value of RRF of 4-95, 4′-95, and 5-95 for X1-95 and 4,5-95 for Y1, Y2, and Y3-95). Statistical analyses were performed by two-way ANOVA with the Bonferroni correction for multiple comparisons, with *p* < 0.05 considered significant. M_WT_, male wildtype; M_KO_, male *Cyp2abfgs*-null; M_KI_, male CYP2A6-humanized; F_WT_, female wildtype; F_KO_, female *Cyp2abfgs*-null; F_KI_, female CYP2A6-humanized mice.

Five mono- and four dihydroxylated metabolites were detected in the intestinal content from all exposure groups (Figure 2; for representative chromatograms, see Figure S3). Five hydroxylated metabolites, including 5-95, 4′-95, 4-95, and 4,5-95, were identified based on authentic standards. These metabolites have been detected previously in serum, tissues, and feces from mice, rats, and quail.^21,22,52,53,66,68^ In addition, one unknown monohydroxylated metabolite, X1-95, and three unknown dihydroxylated metabolites, Y1-95, Y2-95, and Y3-95, were detected. The GC–MS/MS analysis did not allow for the identification of dechlorinated PCB95 metabolites.

Based on the elution order,^66^ the unknown monohydroxylated metabolite, X1-95, likely corresponds to 3′-95. Similarly, the unknown dihydroxylated metabolite, Y1-95, likely corresponds to 3′,4′-95. Finally, Y2-95 and Y3-95 likely are dihydroxylated metabolites that contain the two hydroxy groups in different phenyl rings, as shown in an earlier study of fecal metabolites in mice, rats, and quail.^66^ 4,5-95 was the major metabolite in all intestinal content samples. The rank order of the metabolites based on their average concentrations across all exposure groups was as follows: 4,5-95 > Y1-95 > 4′-95 > 5-95 > X1-95 > 4-95 > Y3-95 > 3-103 > Y2-95 (Figure 2; Figure S2A). The OH-PCB profiles across different exposure groups were similar, with similarity coefficients, cos θ, ranging from 0.92 to 1.00 (Figure S2B).

In contrast to the present study, where 4,5-95 was the major metabolite, 5-95 was the major metabolite in feces from mice and rats exposed orally to PCB95,^66^ an observation that is probably due to differences in the exposure paradigms or sampling time points across studies. Alternatively, differences in the analytical approaches may explain these differences. The earlier study detected complex mixtures of monohydroxylated metabolites, for example, 3′-95, 4′-95, and 4-95, and diOH-PCB95 metabolites in the feces, which agrees with the present study. 5-95 was the major PCB95 metabolite formed in metabolism studies with hepatic microsomes from rats and mice.^65,69^ 4′-95 and 4-95 were minor metabolites formed by mouse liver microsomes.^65^ Metabolism studies with pooled human liver microsomes and recombinant cytochrome P450 enzymes reported the formation of 4′-95 as the major metabolite and other mono- and dihydroxylated metabolites as minor metabolites by CYP2A6.^17,18^ These results underscore the well-documented differences between the species in the metabolism of PCB95.

### Subject Screening for PCB95 Metabolites in the Intestinal Content

**Figure 3:**
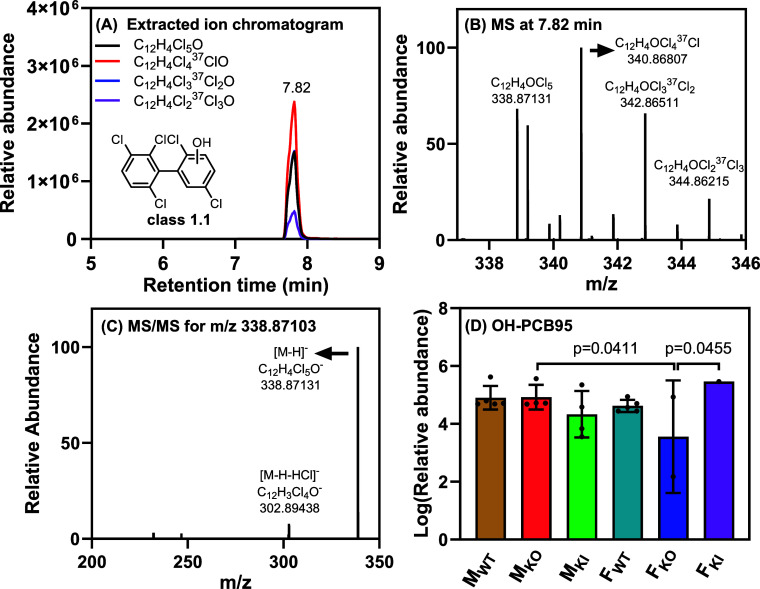
OH-PCB 95 metabolites were the major metabolite detected by LC–HRMS in intestinal content samples of male and female wildtype (WT), *Cyp2abfgs*-null (KO), and CYP2A6-humanized (KI) mice exposed to PCB95 (*n* = 5–7 per exposure group). (A) Representative chromatogram of the four highest abundance isotope ions extracted from the full scan mode, (B) accurate mass spectrum at 7.82 min showing an isotopic pattern of penta-chlorinated compound (62:100:65:21), and (C) parallel reaction monitoring (PRM) spectrum showing the fragment ion of [M – H–HCl]^−^ supporting the identification of the OH-PCB95 metabolite. (D) Relative abundance of the OH-PCB95 in log scale across the exposure groups. The relative abundance values were adjusted by the respective PFOS signal and the sample weight. The LC–HRMS analyses were performed in negative mode. Statistical analyses were performed by two-way ANOVA with the Bonferroni correction for multiple comparisons, with *p* < 0.05 considered significant.

**Figure 4:**
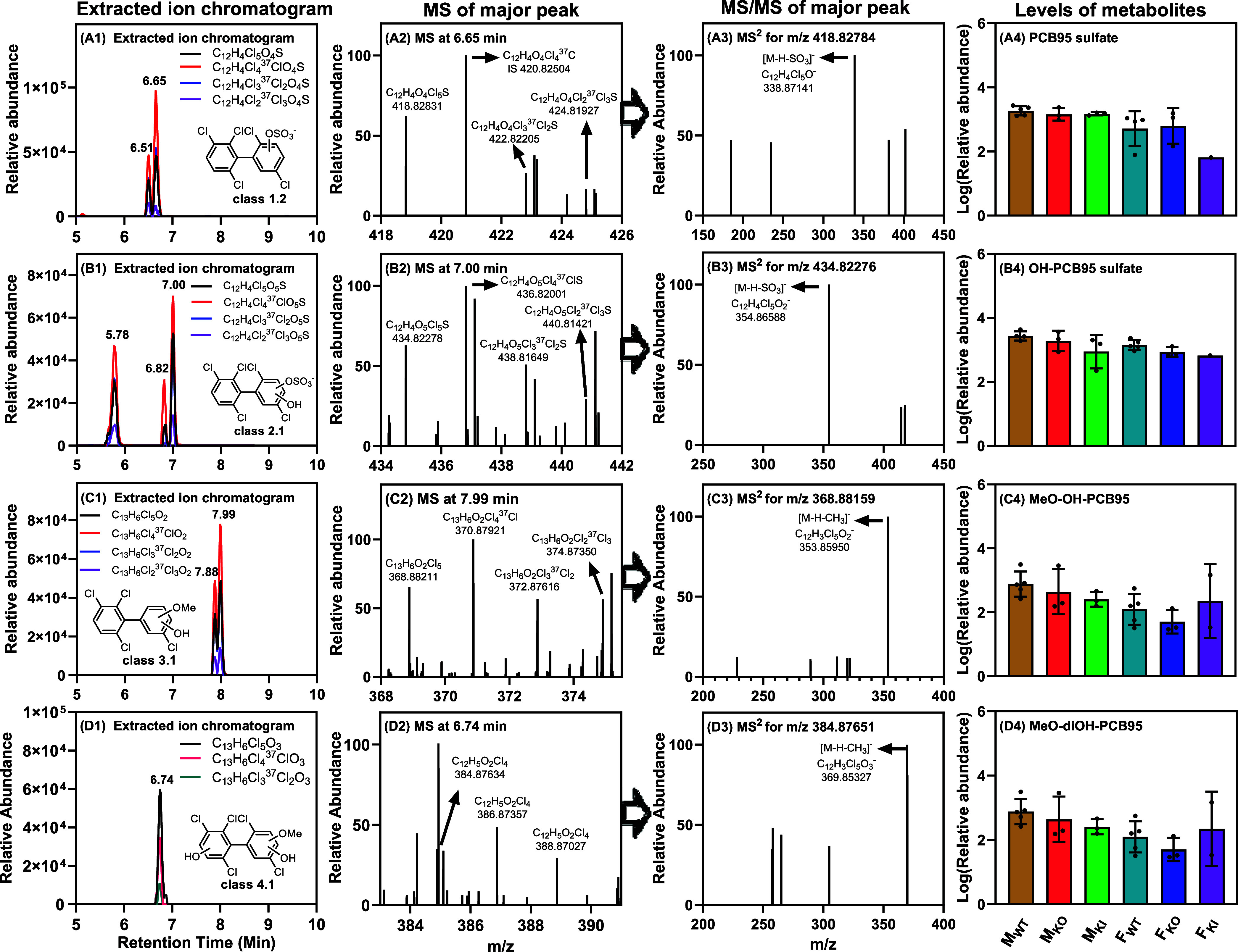
Several PCB95 metabolites, including two PCB95 sulfates (panels A), three OH-PCB95 sulfates (panels B), two MeO–OH-PCB95 (panels C), and one MeO-diOH-PCB95 (panels D), were detected and confirmed in the PRM mode in a representative sample from a male wildtype (WT) mouse exposed to PCB95. Chromatograms (A1–D1) were extracted from the full scan data with the top three or four abundant isotopic ions of PCB95 sulfates (A1), OH-PCB95 sulfates (B1), MeO–OH-PCB95 (C1), and MeO-diOH-PCB95 (D1). Accurate mass spectra, i.e., PCB95 sulfates (A2), OH-PCB95 sulfates (B2), MeO–OH-PCB95 (C2), and MeO-diOH-PCB95 (D2) showed the isotopic pattern of penta-chlorinated compounds. The corresponding PRM fragment mass spectra (A3–D3) supported the identification of the PCB metabolites; i.e., PCB95 sulfates (panels A) and OH-PCB95 sulfates (panels B) were sulfate metabolites, and MeO–OH-PCB95 (panels C) and MeO-diOH-PCB95 (panels D) were methoxylated compounds. The total relative abundance of the metabolites (A4-D4) showed no significant difference between exposure groups. The relative abundance values were adjusted by the respective PFOS signal and the sample weight. The LC–HRMS analyses were performed in negative mode. Statistical analyses were performed by two-way ANOVA with the Bonferroni correction for multiple comparisons, with *p* < 0.05 considered significant. M_WT_, male wildtype; M_KO_, male *Cyp2abfgs*-null; M_KI_, male CYP2A6-humanized; F_WT_, female wildtype; F_KO_, female *Cyp2abfgs*-null; F_KI_, female CYP2A6-humanized mice.

The disposition and toxicity of mono and dihydroxylated PCBs, PCB quinones, and, to a more limited degree, PCB sulfates have received some attention in the scientific literature.^30,32,70^ Much less is known about the disposition and toxicity of other potentially toxic PCB metabolites because they are not amenable to GC–MS/MS analyses, or suitable analytical standards and test compounds are unavailable. PCB conjugates of mono- and, potentially, dihydroxylated PCBs can be identified by GC–MS/MS as the corresponding methoxylated derivatives. However, this approach involves a labor-intensive, multistep workflow using enzymatic deconjugation with purified enzymes.^71^ Here, we used subject screening of intestinal content samples with LC–HRMS to identify eight PCB95 metabolite classes formed in vivo, including several previously unreported metabolite classes of PCB95 (Table 1). We also explored differences in the relative levels of PCB95 metabolites in transgenic animals with genetic manipulations of specific cytochrome P450 genes (Table 1; Figures 3 and 4).

**Table 1:** Eight Classes of PCB Metabolite Were Detected by LC-Orbitrap MS in the Intestinal Content of Mice Exposed to PCB95^c^

class no.	metabolites	RT (RRT)^a^	formula[M – H]^−^	calculated (Da)	measured (Da)	Δmz^b^ (ppm)	MS^2^ (Da)	confidence level
1.1	OH-PCB95	7.77 (1.14)	C_12_H_4_Cl_5_O^–^	338.87103	338.87104	0.02	[M – H–HCl]^−^, 302.89438	2
1.2	PCB95 sulfate	6.51 (0.96)	C_12_H_4_Cl_5_O_4_S^–^	418.82784	418.82768	–0.37	[M – H–SO_3_]^−^, 338.87253	2
		6.67 (0.98)		418.82784	418.82781	–0.07	[M – H–SO_3_]^−^, 338.87141	2
2	OH-PCB95 sulfate	5.78 (0.85)	C_12_H_4_Cl_5_O_5_S^–^	434.82276	434.82301	0.58	[M – H–SO_3_]^−^, 354.86606	2
		6.87 (1.01)		434.82276	434.82303	0.62	[M – H–SO_3_]^−^, 354.86609	2
		7.01 (1.03)		434.82276	434.82349	1.69	[M – H–SO_3_]^−^, 354.86588	2
3	MeO–OH-PCB95	7.88 (1.16)	C_13_H_6_Cl_5_O_2_^–^	368.88159	368.88224	1.77	[M – H–CH_3_]^−^, 353.85785	2
		8.00 (1.18)		368.88159	368.88216	1.56	[M – H–CH_3_]^−^, 353.85950	2
4	MeO-diOH-PCB95	6.73 (0.99)	C_13_H_6_Cl_5_O_3_^–^	384.87651	384.87471	–4.67	[M – H–CH_3_]^−^, 369.85327	2
5	PCB95 sulfonate	5.96 (0.88)	C_12_H_4_Cl_5_O_3_S^–^	402.83293	402.83326	0.81	NA	3
6	OH-tri-CB	7.68 (1.13)	C_12_H_6_Cl_3_O^–^	270.94897	270.94919	0.81	NA	3
7	OH-tetra-CB	6.48 (0.95)	C_12_H_5_Cl_4_O-	304.91000	304.90996	–0.12	NA	3
		7.69 (1.13)		304.91000	304.91001	0.04	NA	3
		7.80 (1.15)		304.91000	304.90996	–0.13	NA	3
8	diOH-tri-CB	7.60 (1.12)	C_12_H_6_Cl_3_O_2_^–^	286.94389	286.94400	0.39	NA	3

a RT indicates the average retention time in min, and RRT indicates the average relative retention time of PCB metabolites to the internal standard (PFOS).

b Difference in the accurate mass between the theoretical and measured values (Δmz) was calculated by the following formula, Δmz = (MZ_measured_ – MZ_theoretical_)/MZ_theoretical_ × 10^6^. The Δmz values were the average values of the same PCB metabolites from all samples.

c For the chemical structures and the corresponding abbreviations of the PCB95 metabolites, see Figure 5.

The first four metabolite classes identified via subject screening are metabolites formed by successive oxidation of PCB95 to mono-, di-, and, ultimately, trihydroxylated metabolites. Class 1 metabolites included one peak corresponding to OH-PCB95 metabolites (Class 1.1; Figure 3) and two PCB95 sulfate conjugates (Class 1.2) (Table 1). The accurate mass spectrum of the molecular ion, [M – H]^−^, showing the characteristic isotope pattern of a penta-chlorinated compound, and the PRM analysis showing the loss of HCl, support the identification of this metabolite at a confidence level of 2 (Figure 3B,C). The five OH-PCB95 metabolites detected in the GC–MS/MS analysis likely coeluted in the LC separation, as observed for monohydroxylated metabolites of PCB3,^27^ or are below the detection limit of the LC–HRMS analysis.

The PCB95 sulfate peaks showed the characteristic isotope pattern on a penta-chlorinated PCB derivative and the loss of SO_3_, as shown for the major peak at 6.65 min (Figure 4A2,A3), supporting the identification of both metabolites as PCB sulfates with confidence levels of 2. PCB95 sulfate metabolites have not been reported previously in vitro or in vivo; however, we noted the presence of complex mixtures of PCB sulfates in the feces of mice exposed to an environmental PCB mixture.^29,35^ Lower chlorinated PCB sulfates have been detected in serum samples from the United States, including a PCB52 sulfate structurally related to PCB95.^71,72^ No PCB95 arene oxide, dihydrodiol, or glucuronide metabolites were detected in intestinal content, consistent with our earlier study of feces from mice exposed to an environmental PCB mixture.^29,35^ Glucuronides of lower chlorinated PCBs are formed by HepG2 cells in culture^34,73^ or present in the intestinal compartment of rat fetuses.^74^

Class 2 and 3 metabolites have a diOH-PCB95 structure (Table 1). Class 2 comprised three hydroxylated sulfate conjugates (OH-PCB95 sulfate). The isotope pattern of the molecular ion and fragment ions indicating the loss of the sulfate group in the PRM spectrum, as shown for the metabolite with a retention time of 7.00 min (Figure 4B2,B3), corroborated the identification of these metabolites at a confidence level of 2. No diOH-PCB95 metabolites were observed, unlike the GC–MS/MS analysis, likely because they were below the detection limit of the LC–HRMS analysis or because they coeluted with the corresponding conjugates, making it impossible to distinguish them from fragment ions.^73^ diOH-PCB95 metabolites have also been reported earlier in feces from rodents and quail.^66^ Although OH-PCB95 sulfates have not been reported previously, other OH-PCB sulfates have been detected in mouse feces following oral exposure to an environmental PCB mixture^29,35^ or mouse serum and liver after acute, oral PCB11 exposure.^75^ A study in rats demonstrates that OH-PCB sulfates are formed by oxidation of the corresponding PCB sulfate administered intravenously.^36^ Alternatively, OH-PCBs may be formed by the sulfation of dihydroxylated PCB metabolites. OH-PCB91 glucuronides have been detected in mouse urine;^67^ however, these metabolites were not detected in this study.

Class 3 metabolites of PCB95 included two methoxylated-hydroxylated PCB95 isomers (MeO–OH-PCB95) (Table 1). The identification of both metabolites was confirmed by the characteristic chlorine isotope pattern on the molecular ion and fragment ions, indicating the loss of a methyl group, as shown for the metabolite at a retention time of 7.99 min (Figure 4C2,C3). Both isomers were identified with a confidence level of 2. Earlier disposition studies in toxicologically relevant animal models also reported the formation of MeO–OH-PCB metabolites.^76−78^ MeO–OH-PCBs were also detected in the feces of mice exposed to an environmental PCB mixture.^29^ No sulfate or glucuronide MeO–OH-PCB95 conjugates were detected in the present study. In contrast, MeO–OH-PCB sulfates were found in the feces of mice exposed to the environmental PCB mixture.^35^ MeO–OH-PCB metabolites of lower chlorinated PCBs and the corresponding sulfate and glucuronide metabolites were also detected in HepG2 cells in culture.^34,73^ Indirect evidence suggests that these MeO–OH-PCBs are catechol metabolites methylated by catechol-*O*-methyltransferase.^79^ Because PCB catechols are highly toxic metabolites,^70^ the methylation of dihydroxylated PCBs likely represents a detoxication pathway.

Class 4 metabolites included one peak corresponding to a methoxylated-diOH-PCB95 metabolite (MeO-diOH-PCB95) (Table 1). The characteristic chlorine isotope pattern of the molecular ion and loss of a methyl group in the PRM spectrum confirmed the identification of this metabolite with a confidence level of 2 (Figure 4D2,D3). No MeO-diOH-PCB95 conjugates were detected in the LC–HRMS analysis. MeO-diOH-PCB metabolites or their conjugates were not detected in feces from mice exposed to an environmental PCB mixture, possibly because of the different PCB doses and dosing paradigms.^29^ However, MeO-diOH-PCB metabolites of lower chlorinated PCBs, including their monosulfated and monoglucuronidated metabolites, were formed in HepG2 cells in culture, a finding that underscores the potential human relevance of these metabolites.^34,73^

Class 5 metabolites included one peak corresponding to a PCB95 sulfonate metabolite (Figure S4), identified with a confidence level of 3 (Table 1). PCB95 sulfonates have not been reported previously; however, various PCB sulfonate metabolites were present in the feces of mice exposed to an environmental PCB mixture^29^ and in serum from polar bears.^35^ These sulfonates are likely formed in a multistep reaction via the mercapturic acid pathway, as reported by earlier studies in mice and rats.^27,80,81^ Recent studies report the presence of hundreds of PCB sulfonates and the corresponding hydroxylated metabolites in soil and plant root samples.^82,83^ Therefore, more research is needed to characterize the occurrence of these metabolites in environmental samples, wildlife, foodstuffs, and humans as well as to assess their toxicity.

Three dechlorinated PCB95 metabolite classes were detected (Table 1), including peaks corresponding to one monohydroxylated trichlorobiphenyl (OH-tri-CB, Class 6; Figure S5), three monohydroxylated tetrachlorobiphenyl (OH-tetra-CB, Class 7; Figure S6), and one dihydroxylated trichlorobiphenyl (diOH-tri-CB, Class 7; Figure S7). These metabolites were identified with a confidence level of 3. Dechlorinated OH-PCB95 metabolites have been previously detected in the feces of rats exposed to PCB95.^66^ Similarly, dechlorinated OH-PCB metabolites were formed from PCB153 in rabbits.^77^ While the presence of diOH-tetra-CBs in feces from rats exposed to PCB95 has been described previously,^66^ no diOH-tetra-CB metabolites were detected in this study. Moreover, structurally similar dechlorinated dihydroxylated PCB metabolites were formed in incubations using HepG2 cells in culture and human liver microsomes.^34^ A recent study reported that dechlorinated OH-PCB metabolites of PCB28 are formed by recombinant human CYP1A2,^84^ findings demonstrating that similar dechlorinated reactions also occur in humans.

### PCB95 Metabolism Pathway in Mice

The present study demonstrates that the characterization of the PCB metabolome in the intestinal content using a combination of analytical approaches (GC–MS/MS and LC–HRMS) allows a comprehensive characterization of the metabolic pathway of PCBs, such as PCB95, in vivo, as shown in Figure 5:

1. PCB95 is initially oxidized by cytochrome P450 enzymes to form a complex mixture of OH-PCB95 metabolites (Class 1.1). This oxidation can involve an arene oxide intermediate that subsequently rearranges to an OH-PCB95 or by direct insertion of an oxygen atom into an aromatic C–H bond.
2. Further metabolism of OH-PCB95 metabolites involves conjugation reactions, particularly sulfation reactions, resulting in the formation of PCB95 sulfates (Class 1.2).
3. OH-PCB95 can also be metabolized by cytochrome P450 enzymes to diOH-PCB95 metabolites.
4. diOH-PCB95 can undergo conjugation reactions, particularly sulfation reactions, resulting in the formation of OH-PCB95 sulfates (Class 2).
5. OH-PCB95 sulfates (Class 2) may also be formed via the oxidation of PCB95 sulfates by cytochrome P450 enzymes.
6. OH-PCB95 metabolites can also undergo successive enhanced reductive dechlorination, resulting in OH-tetra-CB (Class 7) and OH-tri-CB metabolites (Class 6).
7. OH-tetra-CB may undergo further oxidative metabolism, resulting in the formation of a diOH-tri-CB metabolite (Class 8).
8. OH-tri-CB metabolites may be oxidized directly to the diOH-tri-CB metabolite (Class 8).
9. diOH-PCB95, in particular diOH-PCB95 metabolites with vicinal OH groups, can be methylated by COMT, resulting in a MeO–OH-PCB95 metabolite (Class 3).
10. Oxidation of MeO–OH-PCB95 metabolites by cytochrome P450 enzymes results in the formation of a MeO-diOH-PCB95 metabolite (Class 4).
11. PCB95 is also metabolized to PCB95 sulfonates, a PCB metabolism pathway that is poorly investigated but warrants further investigation because of the environmental prevalence of these metabolites.^35,82,83^

**Figure 5:**
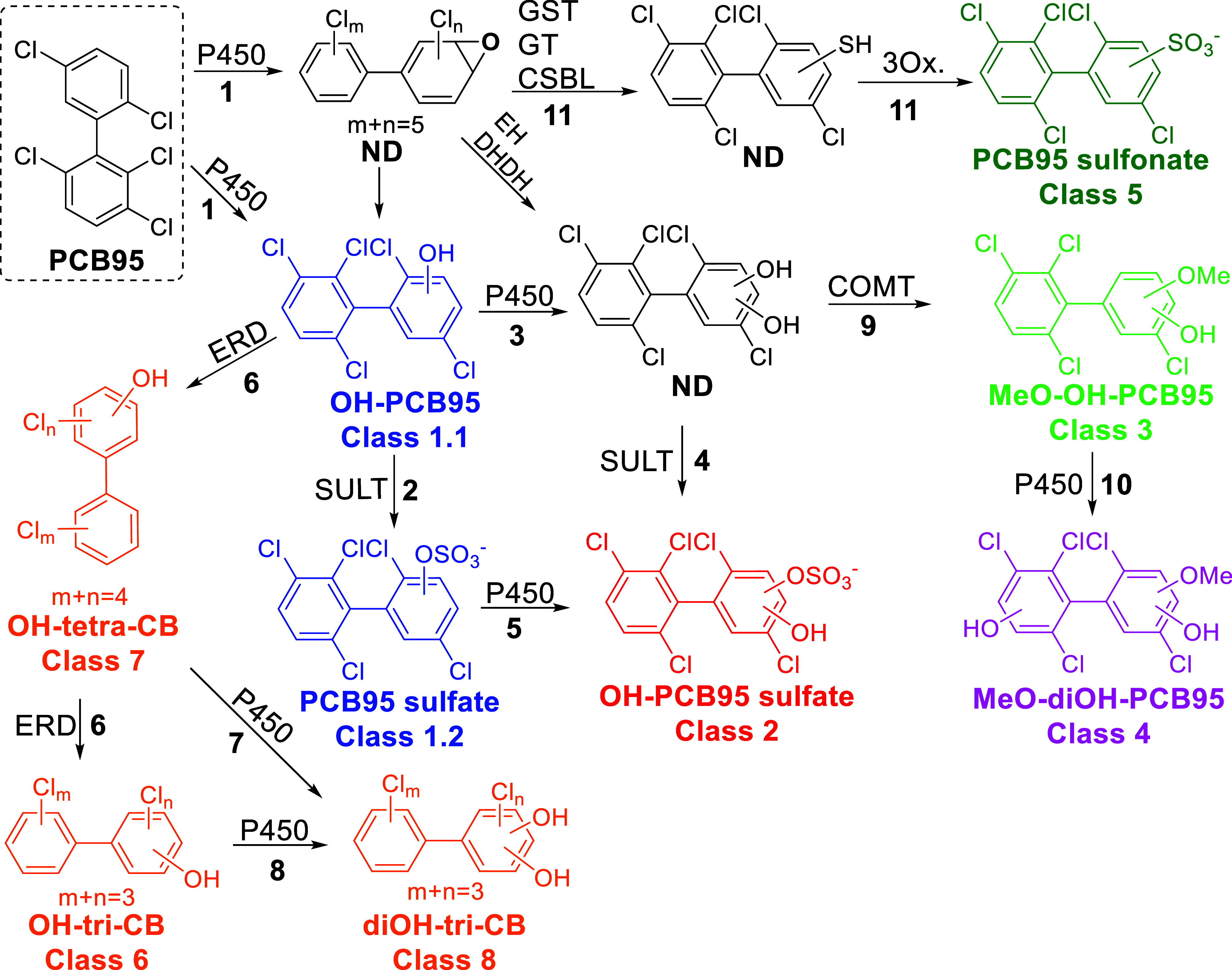
Proposed metabolic pathway of PCB95 in mice exposed to PCB95 based on the LC–HRMS analysis of intestinal content samples. P450: cytochrome P450 enzyme; GST: glutathione-*S*-transferase; GT: gamma-glutamyl transferase; CG: cysteinglycinase; CSBL: cysteine *S*-conjugate beta-lyase; Ox.: oxidation; SULT: sulfotransferase; COMT: catechol-*O*-methyltransferase; EH: epoxide hydrolase; DHDH: dihydrodiol dehydrogenase; ERD: enhanced reductive dechlorination; ND: not detected in this study. The liver serves as the primary site for PCB95 metabolism, though other sites, including the lungs and gut microbiota, may also play a role in PCB metabolism.

While the metabolism pathway for PCB95 is consistent with the general PCB metabolism literature, our results underscore that PCB sulfones and various dechlorinated PCB metabolites are overlooked metabolites that are formed in vivo. More research is warranted to characterize their formation and toxicities in animal models and humans. In addition, it is important to acknowledge that the GC–MS/MS and LC–HRMS analyses may have overlooked additional metabolite classes formed in vivo. For example, both analytical approaches are unlikely to capture methyl sulfone PCB metabolites, a major class of PCB metabolites.^85^

### Effect of Genotype and Sex on PCB95 Metabolite Levels in the Intestinal Content

In addition to elucidating the PCB95 metabolism pathway, we explored whether subject screening of intestinal content can be used to characterize sex or mouse strain differences in PCB95 metabolism. For example, the expression of cytochrome P450 enzymes in mice is sex-dependent, resulting in sex differences in specific OH-PCB metabolites formed in in vitro metabolism studies.^65,86^ Moreover, the deletion of *Cyp2abfgs* in KO mice and the presence of CYP2A6 in KI mice are expected to decrease and increase the level of OH-PCB95 metabolites formed by these cytochrome P450 enzymes, respectively.

Based on the LC–HRMS data, a significant sex difference in the relative OH-PCB95 levels was observed for M_KO_ and F_KO_ (lower in F_KO_, Figure 3D). Moreover, OH-PCB95 levels in F_KO_ mice were lower than those in F_WT_ and F_KI_ mice. However, this difference was statistically significant only when comparing F_KO_ to F_KI_ mice. No significant differences in the OH-PCB95 levels were observed in male mice. Based on the GC–MS/MS analysis, these differences may be due to the X1-95 and Y1-95 (Figure 2). It appears that CYP enzymes other than those encoded by Cyp2abfgs were sufficient in producing these OH-PCB95 metabolites in male, but not female, WT mice and that CYP2A6 was able to compensate for this KO-associated deficiency in female KI mice. The difference in PCB metabolism between sexes and genotypes may be due to variations in sex and growth hormone levels, which regulate the expression and activity of liver CYP enzymes.^49−51,87^ Some sex and metabolite-dependent differences in the formation of OH-PCB metabolites were also observed in earlier metabolism studies using liver microsomes prepared from *Cyp2a(4/5)bgs*-null and *Cyp2f2*-null mice.^65^

In summary, our intestinal content screening approach suggests that the deletion of mouse *Cyp2abfgs* enzymes and the insertion of human CYP2A6 do not significantly affect the metabolism and excretion of OH-PCB95 metabolites as no notable differences by sex or genotype were observed for any PCB95 metabolites (Figure 4A4,B4). While the relative levels of MeOH–OH-PCB95 and MeOH-diOH-PCB95 in the intestinal content of FKO mice were lower compared to those of FWT and FKI mice, these differences were not statistically significant (Figure 4C4,D4). Further research with a larger sample size or the exploration of different time points and PCB95 doses may reveal more pronounced effects in PCB95 metabolism related to sex and genotype.

### Addressing Knowledge Gaps Regarding PCB Disposition and Toxicity Using Subject Screening of Intestinal Content or Feces

Although the metabolism and toxicity of PCBs have been studied extensively for several decades,^30^ the metabolites of PCBs remain understudied, and significant knowledge gaps remain, for example:

1. The metabolic pathways of many PCB congeners, such as PCB95, in wildlife, laboratory animals, and humans remain unexplored.
2. The role of specific drug-metabolizing enzymes, particularly cytochrome P450 enzymes, in the metabolism and toxicity of different PCB congeners needs further elucidation, especially in toxicologically relevant animal models and humans.
3. The toxicity of complex mixtures of PCB metabolites, including PCB sulfates, PCB glucuronides, and methoxylated PCB metabolites, remains largely unexplored.
4. Further research is needed to understand how genetic variations, such as cytochrome P450 enzyme polymorphisms, influence PCB metabolism and the resulting toxicity.
5. The influence of environmental and biological factors, such as diet, microbiome, and coexposure to other xenobiotics, on the metabolism and toxicity of PCBs is not well documented.

As a first step in addressing these knowledge gaps, the subject-screening approach used in this study enabled us to characterize the complex metabolic pathway of PCB95 in mice. It also provided valuable insights into the role (or lack thereof) of specific cytochrome P450 enzymes in the in vivo metabolism of PCB95. We propose that screening of alternative biological matrices, such as intestinal content or feces, and the use of transgenic animal models with altered metabolism, can improve the understanding of PCB metabolism. This approach will enable studies to address the knowledge gaps mentioned above and ultimately facilitate toxicological studies and risk assessments.

## Supporting Information Available

This material is available free of charge via the internet at. The Supporting Information is available free of charge at https://pubs.acs.org/doi/10.1021/acs.chemrestox.4c00350.

Unique identifiers of the analytical PCB and PCB metabolite standards; MRM parameters used for the GC–MS/MS analysis; MDL and LOQ; recoveries of the ongoing precision and recovery standard, surrogate standards in method blanks and extracts from the intestinal content; wet weight adjusted levels of PCB95 and its metabolites and relative abundance of PCB metabolites; p-values from statistical analyses; enantiomeric fractions of PCB95 residues; comparison of OH-PCB profiles; and chromatograms and mass spectra of PCB95 metabolites in the intestinal content of aPCB95-exposed mice (PDF)
