# Supplementary material for: Elucidating the Metabolism of Chiral PCB95 in Wildtype and Transgenic Mouse Models with Altered Cytochrome P450 Enzymes Using Intestinal Content Screening
Source: Chem Res Toxicol. 2024 Nov 19;37(12):1989–2002. doi: 10.1021/acs.chemrestox.4c00350 (PMC11653397; doi:10.1021/acs.chemrestox.4c00350)
Supplement: Supplementary file 1 — tx4c00350_si_001.pdf [file tx4c00350_si_001.pdf]

# SUPPORTING INFORMATION

# Elucidating the Metabolism of Chiral PCB95 in Wildtype and Transgenic Mouse Models with Altered Cytochrome P450 Enzymes Using Intestinal Content Screening

Xueshu Li,[1] Amanda J Bullert,[1,2] Binita Gautam,[1] Weiguo Han,[3] Weizhu Yang,[3] Qing-Yu Zhang,[3] Xinxin Ding,[3] Hans-Joachim Lehmler[1,2,*]

[1] Department of Occupational and Environmental Health, College of Public Health, University of Iowa, Iowa City, IA 52242, USA; [2] Interdisciplinary Graduate Program in Neuroscience, University of Iowa, Iowa City, IA 52242, USA; [3] Department of Pharmacology and Toxicology, College of Pharmacy, University of Arizona, Tucson, Arizona 85721, USA.

**Corresponding Author:**
Hans-Joachim Lehmler
The University of Iowa
Department of Occupational and Environmental Health
University of Iowa Research Park, B164 MTF
Iowa City, IA 52242
Phone: (319) 335-4981
Fax: (319) 335-4290
e-mail: hans-joachim-lehmler@uiowa.edu

**Table of Contents**


**Table S1.** Unique identifiers of the analytical PCB and PCB metabolite standards used in this study. S4

**Table S2.** Multiple reaction monitor (MRM) parameters used for the GC-MS/MS analysis of PCB95 and its metabolites. S6

**Table S3.** Method detection limits (MDL, ng) and limits of quantification (LOQ, ng/g) of the quantification of PCB95 and its metabolites by GC-MS/MS (MRM). S7

**Table S4.** Recoveries of surrogate standards in the GC-MS/MS (MRM) analysis (N=38). S8

**Table S5.** Recoveries of the ongoing precision and recovery (OPR) standard (N=3) n GC-MS/MS (MRM) analyses. S9

**Table S6.** Recoveries rates of surrogate standards in method blanks and extracts from the intestinal content analyzed by LC-HRMS. S10

**Table S7.** Levels (ng/g wet weight) of PCB95 and its metabolites in the intestinal content of male and female wild-type (WT), *Cyp2abfgs*-null (KO), and CYP2A6-transgenic (KI) mice exposed orally to PCB95. S11

**Table S8**. Relative abundance of PCB metabolites in the intestinal content from male and female wild-type (WT), *Cyp2abfgs*-null (KO), and CYP2A6-transgenic (KI) mice exposed to PCB95. S12

**Table S9**. Comparison of p-values for PCB95 and metabolite levels determined by GC-MS/MS in the intestinal content from male (M) and female (F) wild-type (WT), *Cyp2abfgs*-null (KO), and CYP2A6-transgenic (KI) mice exposed to PCB95. S13

**Table S10.** Comparison of p-values for PCB95 and metabolite levels determined by LC-HRMS in the intestinal content from male (M) and female (F) wild-type (WT), *Cyp2abfgs*-null (KO), and CYP2A6-transgenic (KI) mice exposed to PCB95. S14

**Figure S1.** Enantiomeric fractions of PCB95 residues in the intestinal content from male (M) and female (F) wild-type (WT), *Cyp2abfgs*-null (KO), and CYP2A6-transgenic (KI) mice exposed to PCB95. All EF values were significantly lower than the racemic PCB95 standard (EF = 0.498 ±0.004, n=7), corresponding to an enrichment of the (+)-PCB95 (a*S*-PCB95) atropisomer. S15

**Figure S2**. Comparison of mass percentage profiles of OH-PCB metabolites (A) and similarity coefficients in the feces of male and female wildtype, *Cyp2abfgs*-null, and CYP2A6-humanized mice exposed to PCB95. S16

**Figure S3**. Five mono- and four di-hydroxylated metabolites (analyzed as the corresponding methoxylated derivatives) were detected in the intestinal content of a PCB95 exposed female CYP2A6-transgenic ($F_{KI}$) mouse. S17

**Figure S4.** A PCB95 sulfonate metabolite (Class 5, Table 1) was detected by LC-HRMS subject screening in the intestinal content from male (M) and female (F) wild-type (WT), *Cyp2abfgs*-null (KO), and CYP2A6-transgenic (KI) mice exposed to PCB95. S18

**Figure S5**. A trichlorinated OH-PCB metabolite was detected by LC-HRMS subject screening in the intestinal content from male (M) and female (F) wild-type (WT), *Cyp2abfgs*-null (KO), and CYP2A6-transgenic (KI) mice exposed to PCB95. S19

**Figure S6.** Two tetrachlorinated monohydroxylated PCB95 metabolites (OH-tetra-, class 6.2, see Table 1) were detected by LC-HRMS subject screening in the intestinal content from male (M) and female (F) wild-type (WT), *Cyp2abfgs*-null (KO), and CYP2A6-transgenic (KI) mice exposed to PCB95. S20

**Figure S7.** A trichlorinated dihydroxylated PCB95 metabolite (diOH-tri-CB; Class 7, see Table 1) was detected by LC-HRMS subject screening in the intestinal content from male (M) and female (F) wild-type (WT), *Cyp2abfgs*-null (KO), and CYP2A6-transgenic (KI) mice exposed to PCB95. S21

**Reference** S22

**Table S1.** Unique identifiers of the analytical PCB and PCB metabolite standards used in this study.

| Abbreviation | IUPAC Name | FORMULA | Isomeric SMILES | InChI | InChIKey | CAS Registry Number | CAS Registry URL | PubChem CID | PubChem link | DTXSID | Comptox link |
|---|---|---|---|---|---|---|---|---|---|---|---|
| PCB95 | 1,2,4-trichloro-3-(2,5-dichlorophenyl)benzene | C12H5Cl5 | ClC1=C(C2=CC(Cl)=C(OC)C=C2Cl)C(Cl)=CC=C1Cl | InChI=1S/C12H5Cl5/c13-6-1-2-8(14)7(5-6)11-9(15)3-4-10(16)12(11)17/h1-5H | GXNNLIMMEXHBKV-UHFFFAOYSA-N | 38379-99-6 | https://commonchemistry.cas.org/detail?cas_rn=38379-99-6 | 38012 | https://pubchem.ncbi.nlm.nih.gov/compound/38012 | DTXSID3038301 | https://comptox.epa.gov/dashboard/DTXSID3038301 |
| 4-52 | 2,5-dichloro-4-(2,5-dichlorophenyl)phenol | C12H6Cl4O | C1=CC(=C(C=C1Cl)C2=CC(=C(C=C2Cl)O)Cl)Cl | InChI=1S/C12H6Cl4O/c13-6-1-2-9(14)7(3-6)8-4-11(16)12(17)5-10(8)15/h1-5,17H | ZKDSNFDCQYBBIU-UHFFFAOYSA-N | 51274-68-1 | https://commonchemistry.cas.org/detail?cas_rn=51274-68-1 | 39971 | https://pubchem.ncbi.nlm.nih.gov/compound/39971 | DTXSID10199272 | https://comptox.epa.gov/dashboard/DTXSID10199272 |
| 3-103 | 2,4,6-trichloro-3-(2,5-dichlorophenyl)phenol | C12H5Cl5O | ClC1=CC(C2=C(Cl)C=C(Cl)C(O)=C2Cl)=C(Cl)C=C1 | InChI=1S/C12H5Cl5O/c13-5-1-2-7(14)6(3-5)10-8(15)4-9(16)12(18)11(10)17/h1-4,18H | OTDVHFVBSACSKB-UHFFFAOYSA-N | na | na | na | na | na | na |
| PCB117 | 1,2,4,5-tetrachloro-3-(4-chlorophenyl)benzene | C12H5Cl5 | C1=CC(=CC=C1C2=C(C(=CC(=C2Cl)Cl)Cl)Cl)Cl | InChI=1S/C12H5Cl5/c13-7-3-1-6(2-4-7)10-11(16)8(14)5-9(15)12(10)17/h1-5H | ZDDZPDTVCZLFFC-UHFFFAOYSA-N | 68194-11-6 | https://commonchemistry.cas.org/detail?cas_rn=68194-11-6 | 63094 | https://pubchem.ncbi.nlm.nih.gov/compound/63094 | DTXSID9074199 | https://comptox.epa.gov/dashboard/DTXSID9074199 |
| 5-95 | 2,4,5-trichloro-3-(2,5-dichlorophenyl)phenol | C12H5Cl5O | ClC1=CC(C2=C(Cl)C(O)=CC(Cl)=C2Cl)=C(Cl)C=C1 | InChI=1S/C12H5Cl5O/c13-5-1-2-7(14)6(3-5)10-11(16)8(15)4-9(18)12(10)17/h1-4,18H | NGZZCCQHHGJRSN-UHFFFAOYSA-N | na | na | 102344104 | https://pubchem.ncbi.nlm.nih.gov/compound/102344104 | na | na |
| 4'-95 | 2,5-dichloro-4-(2,3,6-trichlorophenyl)phenol | C12H5Cl5O | ClC1=CC(C2=C(Cl)C=CC(Cl)=C2Cl)=C(Cl)C=C1O | InChI=1S/C12H5Cl5O/c13-6-1-2-7(14)12(17)11(6)5-3-9(16)10(18)4-8(5)15/h1-4,18H | GDQBQONNQUYVKG-UHFFFAOYSA-N | na | na | 129853646 | https://pubchem.ncbi.nlm.nih.gov/compound/129853646 | na | na |
| 4-95 | 2,3,5-trichloro-4-(2,5-dichlorophenyl)phenol | C12H5Cl5O | C1=CC(=C(C=C1Cl)C2=C(C=C(C(=C2Cl)Cl)O)Cl)Cl | InChI=1S/C12H5Cl5O/c13-5-1-2-7(14)6(3-5)10-8(15)4-9(18)11(16)12(10)17/h1-4,18H | VLOXUAHEXUHYTO-UHFFFAOYSA-N | na | na | 102344102 | https://pubchem.ncbi.nlm.nih.gov/compound/102344102 | na | na |

**Table S1 (continued).** Unique identifiers of the analytical PCB and PCB metabolite standards used in this study.

| | | | | | | | | | | | |
|---|---|---|---|---|---|---|---|---|---|---|---|
| 4,5-95 | 3,4,6-trichloro-5-(2,5-dichlorophenyl)benzene-1,2-diol | C12H5Cl5O2 | ClC1=CC(C2=C(Cl)C(O)=C(O)C(Cl)=C2Cl)=C(Cl)C=C1 | InChI=1S/C12H5Cl5O2/c13-4-1-2-6(14)5(3-4)7-8(15)10(17)12(19)11(18)9(7)16/h1-3,18-19H | AVAUGFGSPAJGSE-UHFFFAOYSA-N | na | na | 132278490 | https://pubchem.ncbi.nlm.nih.gov/compound/132278490 | na | na |
| PCB204 | 1,2,3,4,5-pentachloro-6-(2,4,6-trichlorophenyl)benzene | C12H2Cl8 | C1=C(C=C(C(=C1Cl)C2=C(C(=C(C(=C2Cl)Cl)Cl)Cl)Cl)Cl)Cl | InChI=1S/C12H2Cl8/c13-3-1-4(14)6(5(15)2-3)7-8(16)10(18)12(20)11(19)9(7)17/h1-2H | JDZUWXRNKHXZFE-UHFFFAOYSA-N | 74472-52-9 | https://commonchemistry.cas.org/detail?cas_rn=74472-52-9 | 91721 | https://pubchem.ncbi.nlm.nih.gov/compound/91721 | DTXSID7074240 | https://comptox.epa.gov/dashboard/DTXSID7074240 |
| 4'-159 | 1,2,3,4-tetrachloro-5-(3,5-dichloro-4-methoxyphenyl)benzene | C13H6Cl6O | COC1=C(C=C(C=C1Cl)C2=CC(=C(C(=C2Cl)Cl)Cl)Cl)Cl | InChI=1S/C13H6Cl6O/c1-20-13-8(15)2-5(3-9(13)16)6-4-7(14)11(18)12(19)10(6)17/h2-4H,1H3 | FGXPVCUIOFCODF-UHFFFAOYSA-N | na | na | 101924076 | https://pubchem.ncbi.nlm.nih.gov/compound/101924076 | na | na |
| 4-PCB52 sulfate | 2,5-dichloro-4-(2,5-dichlorophenyl)phenyl ammonium sulfate | C12H9Cl4NO4S | O=S(OC1=CC(Cl)=C(C2=CC(Cl)=CC=C2Cl)C=C1Cl)(O)=O.N | InChI=1S/C12H6Cl4O4S.H3N/c13-6-1-2-9(14)7(3-6)8-4-11(16)12(5-10(8)15)20-21(17,18)19;/h1-5H,(H,17,18,19);1H3 | DAQCKCZRVSNHHX-UHFFFAOYSA-N | na | na | na | na | na | na |
| 3-F,4'-OH-PCB3 | 4-(3-fluoro-4-chlorophenyl)phenol | C12H8ClFO | C1=CC(=CC=C1C2=CC(=C(C=C2)Cl)F)O | InChI=1S/C12H8ClFO/c13-11-6-3-9(7-12(11)14)8-1-4-10(15)5-2-8/h1-7,15H | DBNVNCYZHMXJJL-UHFFFAOYSA-N | na | na | 20099942 | https://pubchem.ncbi.nlm.nih.gov/compound/20099942 | na | na |
| 3-F,4'-PCB3 sulfate | 4-(3-fluoro-4-chlorophenyl)-phenyl ammonium sulfate | C12H11ClFNO4S | O=S(OC1=CC=C(C2=CC(F)=C(Cl)C=C2)C=C1)(O)=O.N | InChI=1S/C12H8ClFO4S.H3N/c13-11-6-3-9(7-12(11)14)8-1-4-10(5-2-8)18-19(15,16)17;/h1-7H,(H,15,16,17);1H3 | MUYMCEBMJRPJFT-UHFFFAOYSA-N | na | na | na | na | na | na |
| PFOS | potassium;1,1,2,2,3,3,4,4,5,5,6,6,7,7,8,8,8-heptadecafluorooctane-1-sulfonate | C8F17KO3S | C(C(C(C(C(F)(F)S(=O)(=O)[O-])(F)F)(F)F)(F)F)(C(C(C(F)(F)F)(F)F)(F)F)(F)F.[K+] | InChI=1S/C8HF17O3S.K/c9-1(10,3(13,14)5(17,18)7(21,22)23)2(11,12)4(15,16)6(19,20)8(24,25)29(26,27)28;/h(H,26,27,28);/q;+1/p-1 | WFRUBUQWJYMMRQ-UHFFFAOYSA-M | 2795-39-3 | https://commonchemistry.cas.org/detail?cas_rn=2795-39-3 | 23669238 | https://pubchem.ncbi.nlm.nih.gov/compound/23669238 | DTXSID8037706 | https://comptox.epa.gov/dashboard/DTXSID8037706 |

**Table S2.** Multiple reaction monitor (MRM) parameters used for the GC-MS/MS analysis of PCB95 and its metabolites. OH-PCBs were analyzed after derivatization with diazomethane before analysis.

| Abbreviation | Transition (m/z) | Dwell time (ms) | Collision energy (V) |
|---|---|---|---|
| PCB95 | 325.9 → 255.9 | 60 | 30 |
| 4-52 | 321.9 → 279 | 60 | 25 |
| 3-103 | 355.9 → 340.9 | 60 | 10 |
| PCB117 | 325.9 → 255.9 | 60 | 30 |
| X1-95 | 355.9 → 312.9 | 60 | 25 |
| 5-95 | 355.9 → 312.9 | 60 | 25 |
| 4'-95 | 355.9 → 312.9 | 60 | 25 |
| Y1-95 | 385.9 → 343 | 60 | 15 |
| 4-95 | 355.9 → 312.9 | 60 | 25 |
| 4,5-95 | 385.9 → 343 | 60 | 15 |
| Y2-95 | 385.9 → 343 | 60 | 15 |
| Y3-95 | 385.9 → 343 | 60 | 15 |
| PCB204 | 429.8 → 357.9 | 60 | 35 |
| 4'-159 | 389.9 → 374.8 | 60 | 15 |

X1-95 indicates an unknown monohydroxylated PCB95 metabolite; Y1-95, Y2-95, and Y3-95 are unknown dihydroxylated PCB95 metabolites.

**Table S3.** Method detection limits (MDL, ng) and limits of quantification (LOQ, ng/g) of the quantification of PCB95 and its metabolites by GC-MS/MS.

| Analytes | MDL (ng, N=8) | LOQ (ng/g, N=6) |
|---|---|---|
| PCB95 | 0.01 | 1 |
| 3-103 | 0.005 | 0.3 |
| X1-95 | 0.004 | 1 |
| 5-95 | 0.01 | 2 |
| 4'-95 | 0.02 | 3 |
| 4-95 | 0.01 | 1 |
| Y1-95 | 0.04 | 5 |
| 4,5-95 | 0.02 | 4 |
| Y2-95 | 0.002 | 0.05 |
| Y3-95 | 0.002 | 0.03 |

Method detection limit, which was calculated from the method blanks samples with the following formula, $MDL = \overline{x_b} + k \times s_b$, where $\overline{x_b}$ indicates the mean of method blank measurement, $k$ indicates the single tailed Students' t-value appropriate for 99$^{th}$ percentile confident level with a n-1 degree of freedom, and $s_b$ indicates the sample standard deviation of the replicated method blank sample analyses. Similarly, limit of quantification, LOQ, was calculated from the measures of tissue blanks with the formula, $LOQ = \overline{x_{CTL}} + k \times s_b$, where $\overline{x_{CTL}}$ indicate the mean of measurement from the control tissue, $k$ indicates the single tailed Students' t-value appropriate for 99$^{th}$ percentile confident level with a n-1 degree of freedom, and $s_b$ is the corresponding standard deviation.

X1-95 indicates an unknown monohydroxylated PCB95 metabolite; Y1-95, Y2-95, and Y3-95 are unknown dihydroxylated PCB95 metabolites.

**Table S4.** Recoveries of surrogate standards in the GC-MS/MS (MRM) analysis (N=38).

| Analytical standard | PCB117 | 4'-159 | 4-52[#] |
|---|---|---|---|
| **Mean** | 83% | 134% | 63% |
| **SD** | 16% | 14% | 12% |
| **RSD** | 19% | 11% | 19% |

[#] Recovery of 4-52 from 4-PCB52 sulfate spiked to the intestinal content samples before deconjugation.

**Table S5.** Recoveries of the ongoing precision and recovery (OPR) standard (N=3) n GC-MS/MS (MRM) analyses.

| **Analytical standard** | **PCB117** | **4'-159** | **4-52** | **PCB95** | **3-103** | **5-95** | **4'-95** | **4-95** | **4,5-95** |
|---|---|---|---|---|---|---|---|---|---|
| **Mean** | 90% | 130% | 73% | 65% | 83% | 78% | 106% | 107% | 129% |
| **SD** | 18% | 16% | 15% | 4% | 18% | 3% | 7% | 14% | 10% |
| **RSD** | 20% | 12% | 20% | 7% | 21% | 3% | 7% | 13% | 7% |

**Table S6.** Recoveries of surrogate standards in method blanks and extracts from the intestinal content analyzed by LC-HRMS.

| **Matrix** | | **F-PCB-sulfate** | **F-OH-PCB** |
|---|---|---|---|
| **Method blanks (N=6)** | **Mean** | 65% | 63% |
| | **SD** | 15% | 15% |
| | **RSD** | 23% | 24% |
| **Intestinal content extracts (N=30)** | **Mean** | 61% | 39% |
| | **SD** | 13% | 17% |
| | **RSD** | 21% | 44% |

**Table S7.** Levels (ng/g wet weight) of PCB95 and its metabolites in the intestinal content of male and female wild-type (WT), *Cyp2abfgs*-null (KO), and CYP2A6-transgenic (KI) mice exposed orally to PCB95.

| Analytes | $M_{WT}$ (n=6) | $M_{KO}$ (n=7) | $M_{KI}$ (n=6) | $F_{WT}$ (n=7) | $F_{KO}$ (n=7) | $F_{KI}$ (n=5) |
|---|---|---|---|---|---|---|
| **PCB95** | 39±40 | 139±265 | 33±48 | 26±18 | 26±20 | 110±95 |
| **3-103** | 7±4 | 6±2 | 6±6 | 14±6 | 14±7 | 17±11 |
| **X1-95** | 179±143 | 156±57 | 150±143 | 219±116 | 127±112 | 521±475 |
| **5-95** | 424±279 | 333±123 | 300±256 | 544±295 | 359±147 | 934±790 |
| **4'-95** | 452±269 | 447±159 | 298±240 | 356±192 | 414±254 | 764±831 |
| **4-95** | 238±134 | 177±68 | 160±134 | 248±109 | 192±70 | 434±352 |
| **Y1-95** | 463±192 | 363±185 | 187±117 | 323±149 | 127±98 | 523±442 |
| **4,5-95** | 1406±574 | 1034±507 | 570±376 | 1088±492 | 527±255 | 1666±1293 |
| **Y2-95** | 5±2 | 4±3 | 2±1 | 5±4 | 3±2 | 8±8 |
| **Y3-95** | 14±6 | 13±7 | 11±5 | 14±9 | 13±5 | 23±21 |
| **∑OH-PCBs** | 3188±1437 | 2533±915 | 1685±1150 | 2812±1314 | 1774±651 | 4890±4085 |

Abbreviations and animal size: $M_{WT}$, male wildtype (n=6); $M_{KO}$, male knockout (n=7); $M_{KI}$, male knock-in (n=6); $F_{WT}$, female wildtype (n=7); $F_{KO}$, female knockout (n=7); $F_{KI}$, female knock-in (n=5).

**Table S8**. Relative abundance of PCB metabolites in the intestinal content from male and female wild-type (WT), *Cyp2abfgs*-null (KO), and CYP2A6-transgenic (KI) mice exposed to PCB95.[#]

| Class No. | Metabolites | $M_{WT}$ (n=6) | $M_{KO}$ (n=7) | $M_{KI}$ (n=6) | $F_{WT}$ (n=7) | $F_{KO}$ (n=7) | $F_{KI}$ (n=5) |
|---|---|---|---|---|---|---|---|
| 1.1 | OH-PCB95 | 127075±165408(n=5) | 129083±156155(n=4) | 68239±104946(n=4) | 46176±24933(n=5) | 42763±60263(n=2) | 291973(n=1) |
| 1.2 | PCB95 sulfate | 1942±616(n=5) | 1547±723(n=3) | 1497±159(n=3) | 746±454(n=4) | 942±722(n=3) | 65(n=1) |
| 2 | OH-PCB95 sulfate | 2866±998(n=4) | 2226±1398(n=3) | 1261±930(n=3) | 1506±537(n=5) | 897±334(n=3) | 664(n=1) |
| 3 | MeO-OH-PCB95 | 3755±4047(n=5) | 4251±5256(n=3) | 1197±1176(n=3) | 849±640(n=5) | 370±217(n=3) | 2643±2046(n=2) |
| 4 | MeO-diOH-PCB95 | 1099±1168(n=5) | 1079±1568(n=3) | 283±157(n=3) | 199±211(n=5) | 65±58(n=3) | 748±1011(n=2) |
| 5 | PCB95 sulfonate | 99±47(n=4) | 94±43(n=3) | 47±5(n=2) | 61±37(n=3) | 94(n=1) | 22(n=1) |
| 6 | OH-tri-CB | 184±215(n=6) | 128±87(n=3) | 119±81(n=2) | 112±40(n=5) | 137±121(n=3) | 420±372(n=2) |
| 7 | OH-tetra-CB | 930±885(n=6) | 7396±13462(n=4) | 1429±780(n=4) | 1246±1074(n=5) | 667±170(n=3) | 870±47(n=2) |
| 8 | diOH-tri-CB | 101±86(n=4) | 1680±3303(n=4) | 305±306(n=4) | 352±364(n=5) | 223±248(n=3) | 285±29(n=2) |
| sum | ∑OH-PCBs | 114724±163662(n=6) | 145339±157831(n=4) | 73249±106212(n=4) | 51099±25352(n=5) | 31841±50113(n=3) | 151390±210566(n=2) |

[#]The relative abundance values were from the area integration of the chromatogram extracted from the full scan chromatogram with the theoretical values of the top isotopic *m/z* values. These relative abundance values were adjusted by the relative abundance of per ng PFOS (as an internal standard) and the wet weight of fecal samples used for analysis. Abbreviations and animal size: $M_{WT}$, male wildtype (n=6); $M_{KO}$, male knockout (n=7); $M_{KI}$, male knock-in (n=6); $F_{WT}$, female wildtype (n=7); $F_{KO}$, female knockout (n=7); $F_{KI}$, female knock-in (n=5).

**Table S9**. Comparison of p-values for PCB95 and metabolite levels determined by GC-MS/MS in the intestinal content from male (M) and female (F) wild-type (WT), *Cyp2abfgs*-null (KO), and CYP2A6-transgenic (KI) mice exposed to PCB95. The analyses were performed by two-way ANOVA with the Bonferroni test in GraphPad Prism 9.4.

| Analytes | $M_{WT}$ vs. $M_{KO}$ | $M_{WT}$ vs. $M_{KI}$ | $M_{KO}$ vs. $M_{KI}$ | $F_{WT}$ vs. $F_{KO}$ | $F_{WT}$ vs. $F_{KI}$ | $F_{KO}$ vs. $F_{KI}$ | $M_{WT}$ vs. $F_{WT}$ | $M_{KO}$ vs. $F_{KO}$ | $M_{KI}$ vs. $F_{KI}$ |
|---|---|---|---|---|---|---|---|---|---|
| **PCB95** | >0.9999 | >0.9999 | 0.0451 | >0.9999 | 0.1741 | 0.1534 | >0.9999 | 0.1705 | 0.044 |
| **3-103** | >0.9999 | >0.9999 | >0.9999 | >0.9999 | >0.9999 | >0.9999 | >0.9999 | >0.9999 | 0.149 |
| **X1-95** | >0.9999 | >0.9999 | >0.9999 | 0.1213 | >0.9999 | 0.0017 | >0.9999 | 0.6642 | 0.0767 |
| **5-95** | >0.9999 | >0.9999 | >0.9999 | >0.9999 | >0.9999 | >0.9999 | >0.9999 | >0.9999 | 0.2383 |
| **4'-95** | >0.9999 | >0.9999 | >0.9999 | >0.9999 | >0.9999 | >0.9999 | >0.9999 | >0.9999 | >0.9999 |
| **4-95** | >0.9999 | >0.9999 | >0.9999 | >0.9999 | >0.9999 | >0.9999 | >0.9999 | >0.9999 | 0.3213 |
| **Y1-95** | >0.9999 | 0.263 | >0.9999 | 0.0619 | >0.9999 | 0.0241 | >0.9999 | 0.03 | 0.7023 |
| **4,5-95** | >0.9999 | 0.2308 | >0.9999 | >0.9999 | >0.9999 | 0.4782 | >0.9999 | >0.9999 | 0.4199 |
| **Y2-95** | >0.9999 | >0.9999 | >0.9999 | 0.5085 | >0.9999 | 0.4726 | >0.9999 | >0.9999 | 0.9972 |
| **Y3-95** | >0.9999 | >0.9999 | >0.9999 | >0.9999 | >0.9999 | >0.9999 | >0.9999 | >0.9999 | >0.9999 |
| **∑OH-PCB** | >0.9999 | >0.9999 | >0.9999 | >0.9999 | >0.9999 | >0.9999 | >0.9999 | >0.9999 | 0.4496 |

Abbreviations and animal size: $M_{WT}$, male wildtype (n=6); $M_{KO}$, male knockout (n=7); $M_{KI}$, male knock-in (n=6); $F_{WT}$, female wildtype (n=7); $F_{KO}$, female knockout (n=7); $F_{KI}$, female knock-in (n=5).

**Table S10.** Comparison of p-values for PCB95 and metabolite levels determined by LC-HRMS in the intestinal content from male (M) and female (F) wild-type (WT), *Cyp2abfgs*-null (KO), and CYP2A6-transgenic (KI) mice exposed to PCB95. The analyses were performed by two-way ANOVA with the Bonferroni test in GraphPad Prism 9.4.

| **Metabolites** | **$M_{WT}$ vs. $M_{KO}$** | **$M_{WT}$ vs. $M_{KI}$** | **$M_{KO}$ vs. $M_{KI}$** | **$F_{WT}$ vs. $F_{KO}$** | **$F_{WT}$ vs. $F_{KI}$** | **$F_{KO}$ vs. $F_{KI}$** | **$M_{WT}$ vs. $F_{WT}$** | **$M_{KO}$ vs. $F_{KO}$** | **$M_{KI}$ vs. $F_{KI}$** |
|---|---|---|---|---|---|---|---|---|---|
| OH-PCB95 | >0.9999 | >0.9999 | >0.9999 | 0.2246 | >0.9999 | 0.0455 | >0.9999 | 0.0411 | 0.7811 |
| PCB95 sulfate | >0.9999 | >0.9999 | >0.9999 | >0.9999 | >0.9999 | >0.9999 | >0.9999 | >0.9999 | 0.3635 |
| OH-PCB95 sulfate | >0.9999 | >0.9999 | >0.9999 | >0.9999 | >0.9999 | >0.9999 | >0.9999 | >0.9999 | >0.9999 |
| MeO-OH-PCB95 | >0.9999 | >0.9999 | >0.9999 | >0.9999 | >0.9999 | >0.9999 | >0.9999 | 0.6175 | >0.9999 |
| MeO-diOH-PCB95 | >0.9999 | >0.9999 | >0.9999 | >0.9999 | >0.9999 | >0.9999 | 0.2628 | 0.4067 | >0.9999 |
| PCB95 sulfonate | >0.9999 | >0.9999 | >0.9999 | >0.9999 | >0.9999 | >0.9999 | >0.9999 | >0.9999 | >0.9999 |
| OH-tri-CB | >0.9999 | >0.9999 | >0.9999 | >0.9999 | >0.9999 | >0.9999 | >0.9999 | >0.9999 | >0.9999 |
| OH-tetra-CB | >0.9999 | >0.9999 | >0.9999 | >0.9999 | >0.9999 | >0.9999 | >0.9999 | >0.9999 | >0.9999 |
| diOH-tri-CB | >0.9999 | >0.9999 | >0.9999 | >0.9999 | >0.9999 | >0.9999 | >0.9999 | >0.9999 | >0.9999 |
| ∑OH-PCB | >0.9999 | >0.9999 | >0.9999 | 0.9206 | >0.9999 | >0.9999 | >0.9999 | 0.1342 | >0.9999 |

Abbreviations and animal size: $M_{WT}$, male wildtype (n=6); $M_{KO}$, male knockout (n=4); $M_{KI}$, male knock-in (n=4); $F_{WT}$, female wildtype (n=5); $F_{KO}$, female knockout (n=3); $F_{KI}$, male knock-in (n=2).

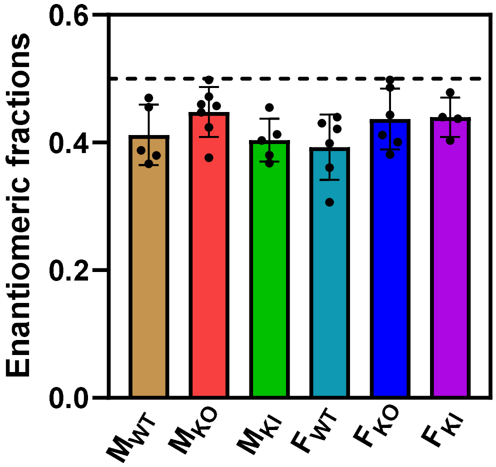


| EF values | |
|---|---|
| $M_{WT}$ (n=5) | 0.41±0.05 |
| $M_{KO}$ (n=7) | 0.45±0.04 |
| $M_{KI}$ (n=5) | 0.40±0.03 |
| $F_{WT}$ (n=6) | 0.39±0.05 |
| $F_{KO}$ (n=6) | 0.44±0.05 |
| $F_{KI}$ (n=4) | 0.44±0.03 |

**Figure S1.** Enantiomeric fractions (EF) of PCB95 residues in the intestinal content from male (M) and female (F) wild-type (WT), *Cyp2abfgs*-null (KO), and CYP2A6-transgenic (KI) mice exposed to PCB95. All EF values were significantly lower than the racemic PCB95 standard (EF = 0.498 ±0.004, n=7), corresponding to an enrichment of the (+)-PCB95 (a*S*-PCB95) atropisomer. The EF values were calculated using the formula: $EF=Area_{E1}/(Area_{E1}+Area_{E2})$.[1]

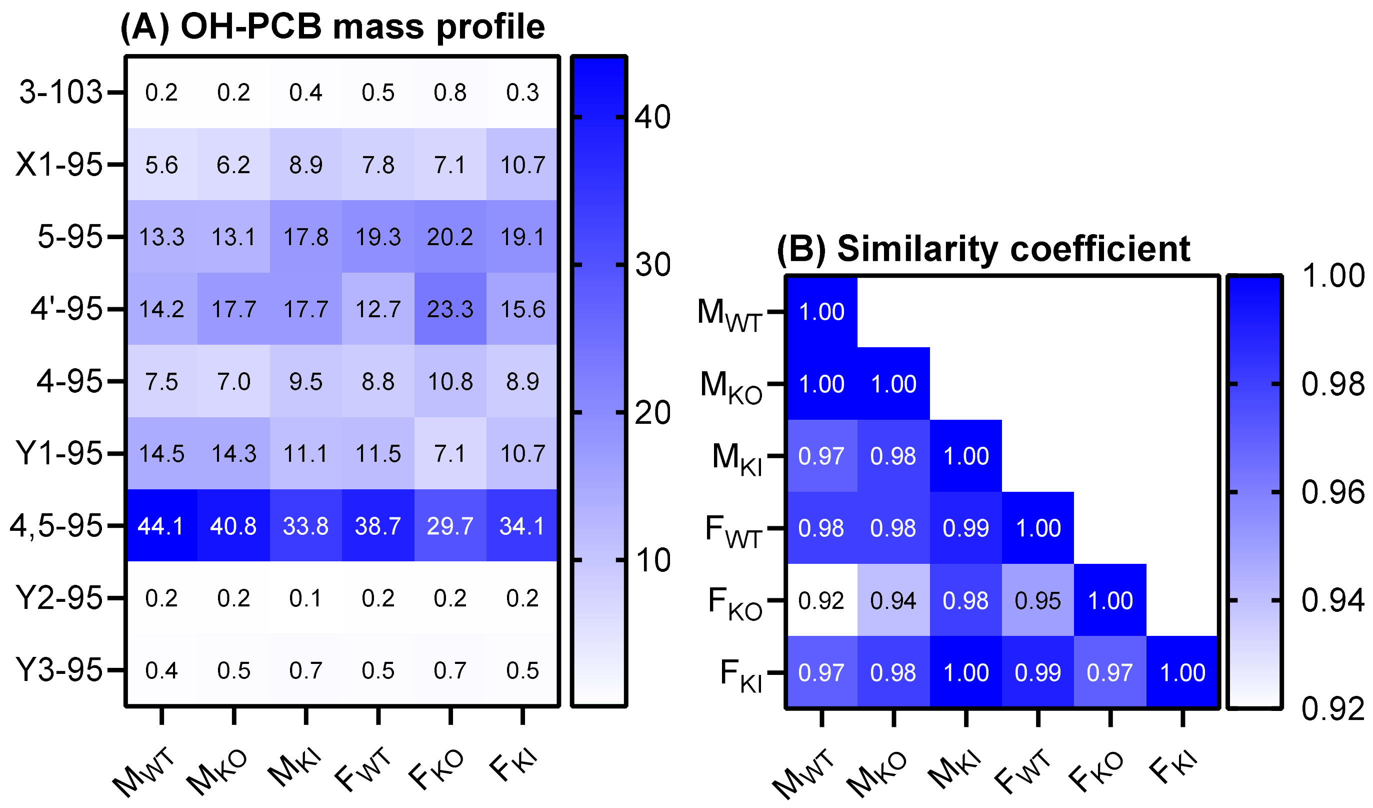


**Figure S2**. Comparison of (A) the mass percentage profiles of OH-PCB metabolites and (B) the similarity coefficients comparing the OH-PCB profiles in the intestinal content from male (M) and female (F) wild-type (WT), *Cyp2abfgs*-null (KO), and CYP2A6-transgenic (KI) mice exposed to PCB95. For a definition of the abbreviations of the PCB metabolites, see Table S1.

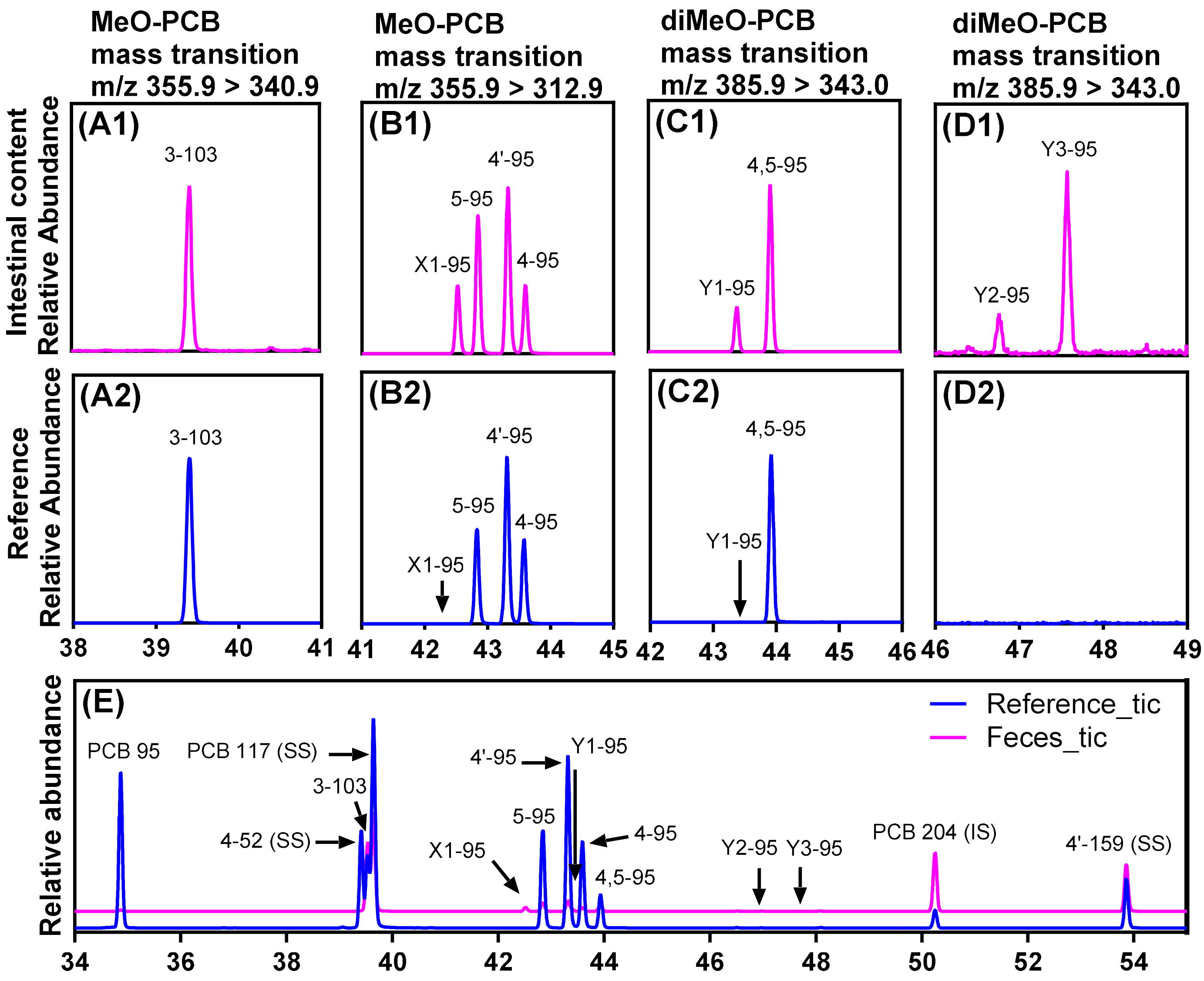


**Figure S3**. Five mono- and four di-hydroxylated metabolites (analyzed as the corresponding methoxylated derivatives) were detected in the intestinal content of a PCB95 exposed female CYP2A6-transgenic ($F_{KI}$) mouse. The extracted ion chromatograms for methoxylated (MeO-PCBs) and dimethoxylated PCBs (diMeO-PCBs) in the intestinal content sample (A1-D1) compared to a reference sample (A2-D2). The extracted chromatograms show mono-hydroxylated PCB95 metabolites with mass transition of *m/z* 355.9 > 340.9 (panels A1 and A2) and *m/z* 355.9 > 312.9 (panels B1 and B2) and di-hydroxylated PCB95 metabolites with mass transition of *m/z* 385.9 > 343.0 (panels C1 and C2, D1 and D2). (E) The total ion chromatogram (TIC) of the reference standard (blue) and the intestinal content sample (pink) shows the presence of PCB95, 3-103, X1-95, 5-95, 4'-95, 4-95, Y1-95, and 4,5-95. Analyses were performed by GC-MS/MS, as described in the Experimental Section. IS, internal standard (PCB204); SS, surrogate standards (PCB117, 4'-159); X1-95, unknown mono-hydroxylated PCB95 metabolite; Y1-95, Y2-95, and Y3-95, unidentified di-hydroxylated PCB95 metabolites.

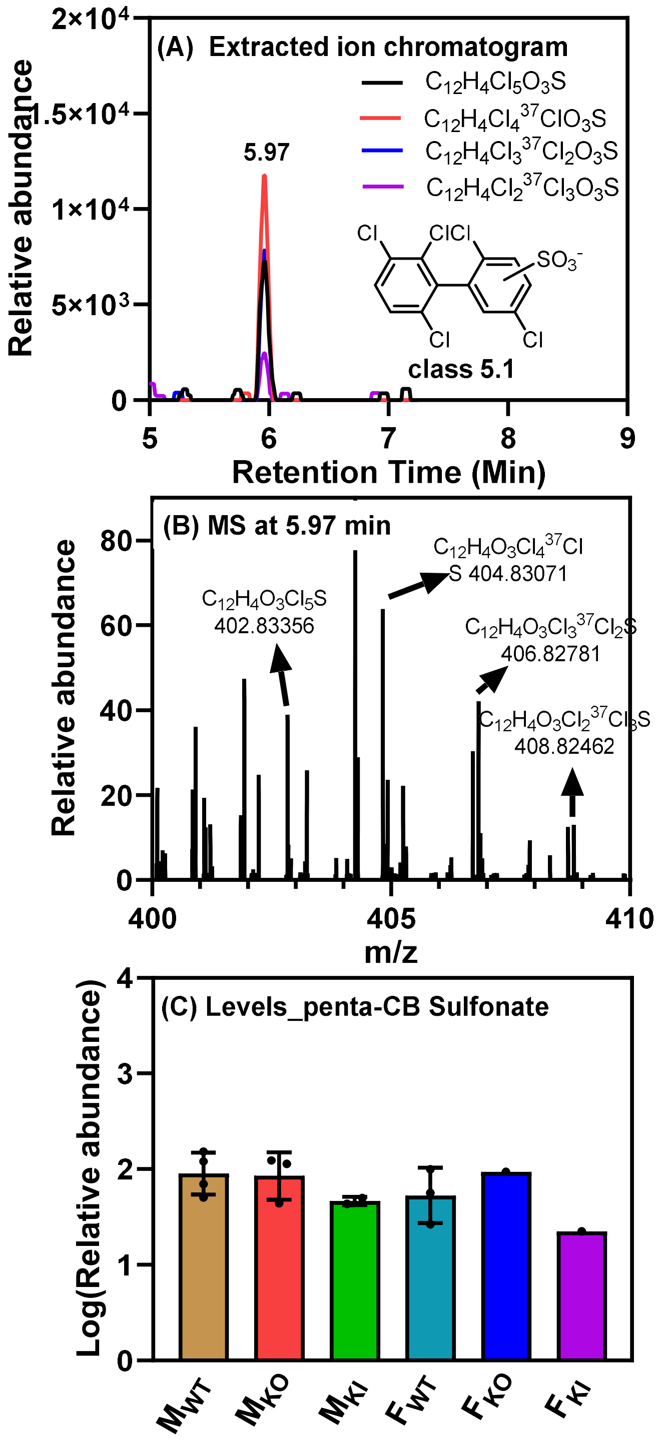


**Figure S4.** A PCB95 sulfonate metabolite (Class 5, **Table 1**) was detected by LC-HRMS subject screening in the intestinal content from male (M) and female (F) wild-type (WT), *Cyp2abfgs*-null (KO), and CYP2A6-transgenic (KI) mice exposed to PCB95. (A) Chromatograms extracted based on the theoretical accurate mass of the top four high-abundance isotope ions of a PCB sulfonate ($[C_{12}H_4O_3Cl_5]^-$, *m/z* 402.83293 for the monoisotopic ion) show a peak corresponding to a PCB95 sulfonate at 5.97 min. (B) The accurate mass of high-abundance isotope ions at 5.97 min matched the theoretical accurate mass and isotopic pattern of a penta-chlorinated compound. (C) The relative abundance of the PCB95 sulfonate showed no significant difference between the exposure groups. The LC-HRMS analysis was performed in the negative polarity mode, as described in the Experimental Section.

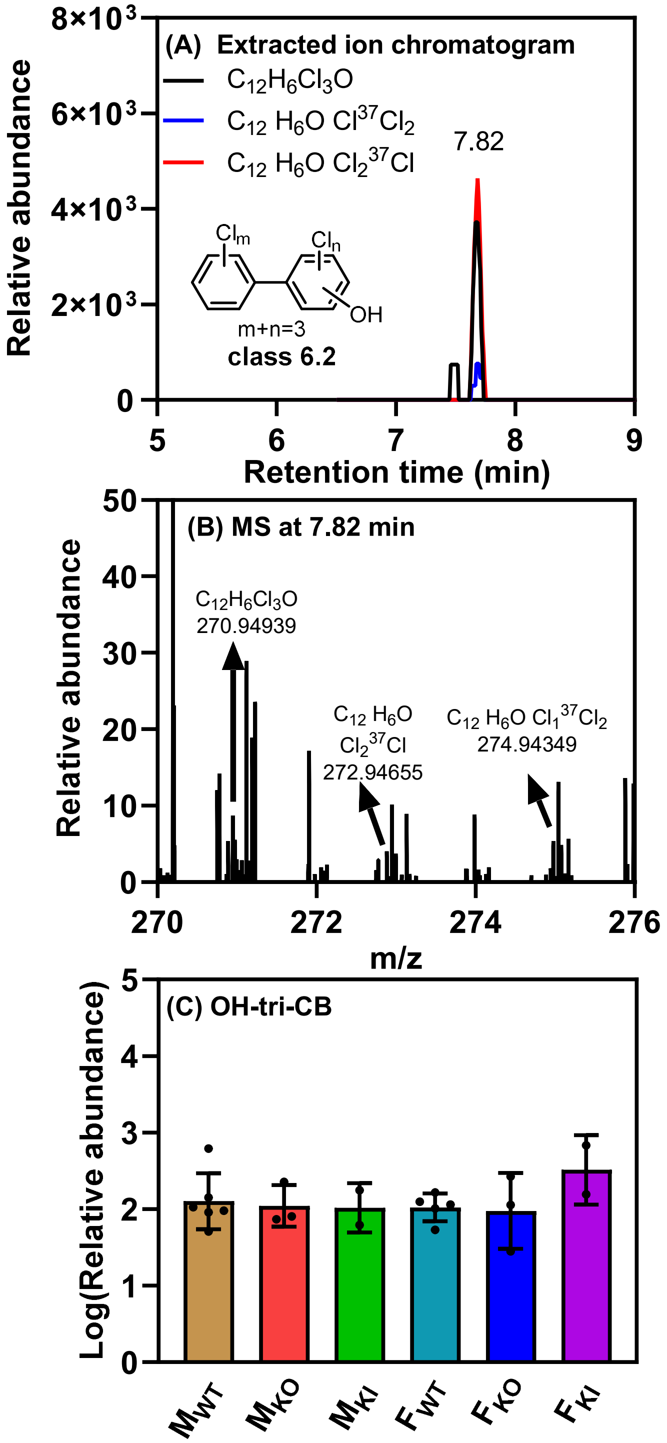


**Figure S5**. A trichlorinated monohydroxylated PCB95 metabolite (OH-tri-CB, Class 6.1, **Table 1**) was detected by LC-HRMS subject screening in the intestinal content from male (M) and female (F) wild-type (WT), *Cyp2abfgs*-null (KO), and CYP2A6-transgenic (KI) mice exposed to PCB95. (A) Chromatograms extracted based on the theoretical accurate mass of the top three high-abundance isotope ions of OH-tri-CBs ($[C_{12}H_6Cl_3O]^-$, *m/z* 270.94897 for the monoisotopic ion) show a peak at 7.82 min. (B) The accurate mass of high-abundance isotope ions at 7.82 min matched the theoretical accurate mass and isotopic pattern of a trichlorinated compound. (C) The relative abundance of OH-tri-CB metabolites showed no significant difference between the exposure groups. The LC-HRMS analysis was performed in the negative polarity mode, as described in the Experimental Section.

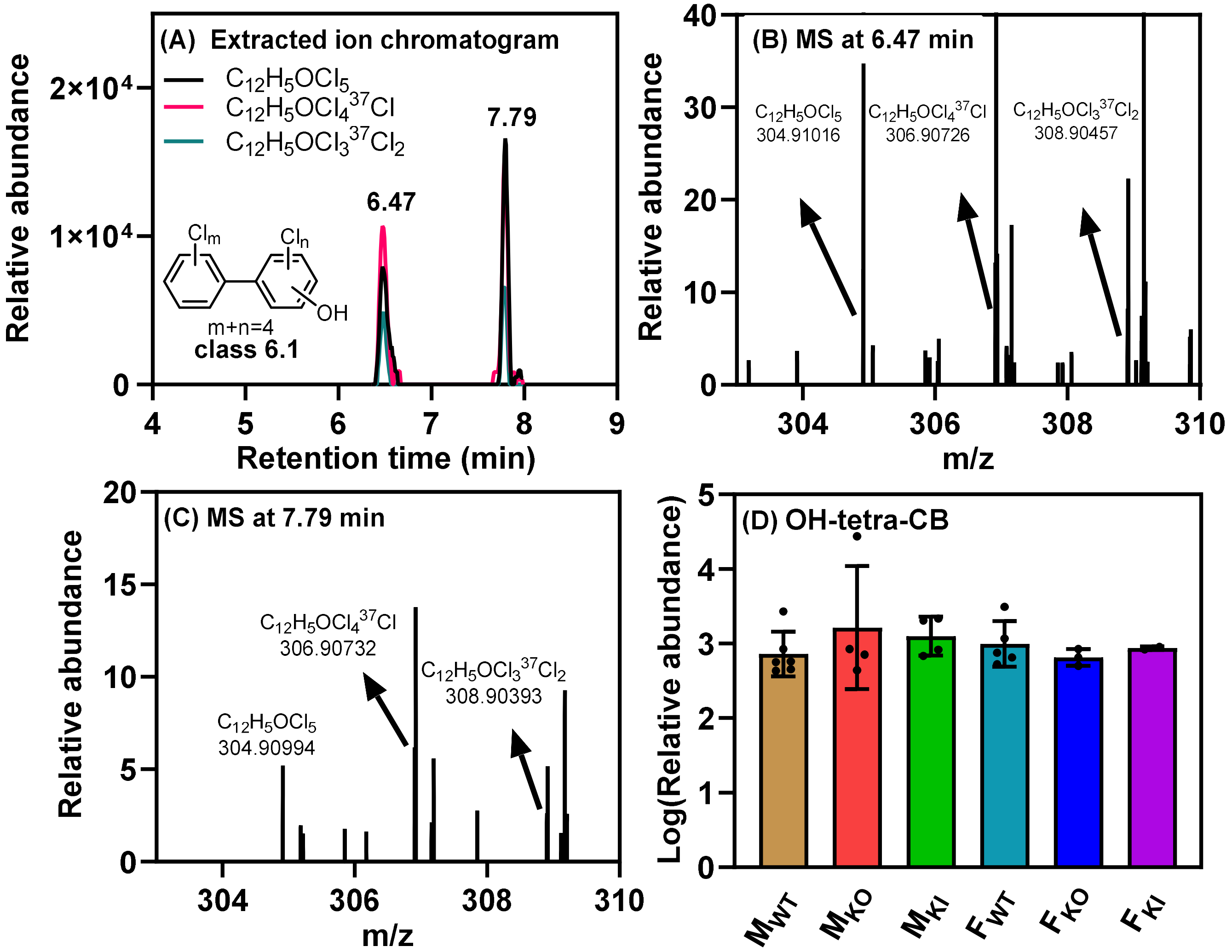


**Figure S6.** Two tetrachlorinated monohydroxylated PCB95 metabolites (OH-tetra-, Class 6.2, **Table 1**) were detected by LC-HRMS subject screening in the intestinal content from male (M) and female (F) wild-type (WT), *Cyp2abfgs*-null (KO), and CYP2A6-transgenic (KI) mice exposed to PCB95. (A) Chromatograms extracted based on the theoretical accurate mass of the top three high-abundance isotope ions of OH-tetra-CBs ($[C_{12}H_5Cl_4O]^-$, *m/z* 304.91000 for the monoisotopic ion) show peaks at 6.47 and 7.79 min. The accurate masses of four high-abundance isotope ions at (B) 6.47 and (C) 7.79 min matched the theoretical accurate mass and isotopic pattern of a tetrachlorinated compound. (D) The relative abundance of OH-tetra-CB metabolites showed no significant difference between the exposure groups. The LC-HRMS analysis was performed in the negative polarity mode, as described in the Experimental Section.

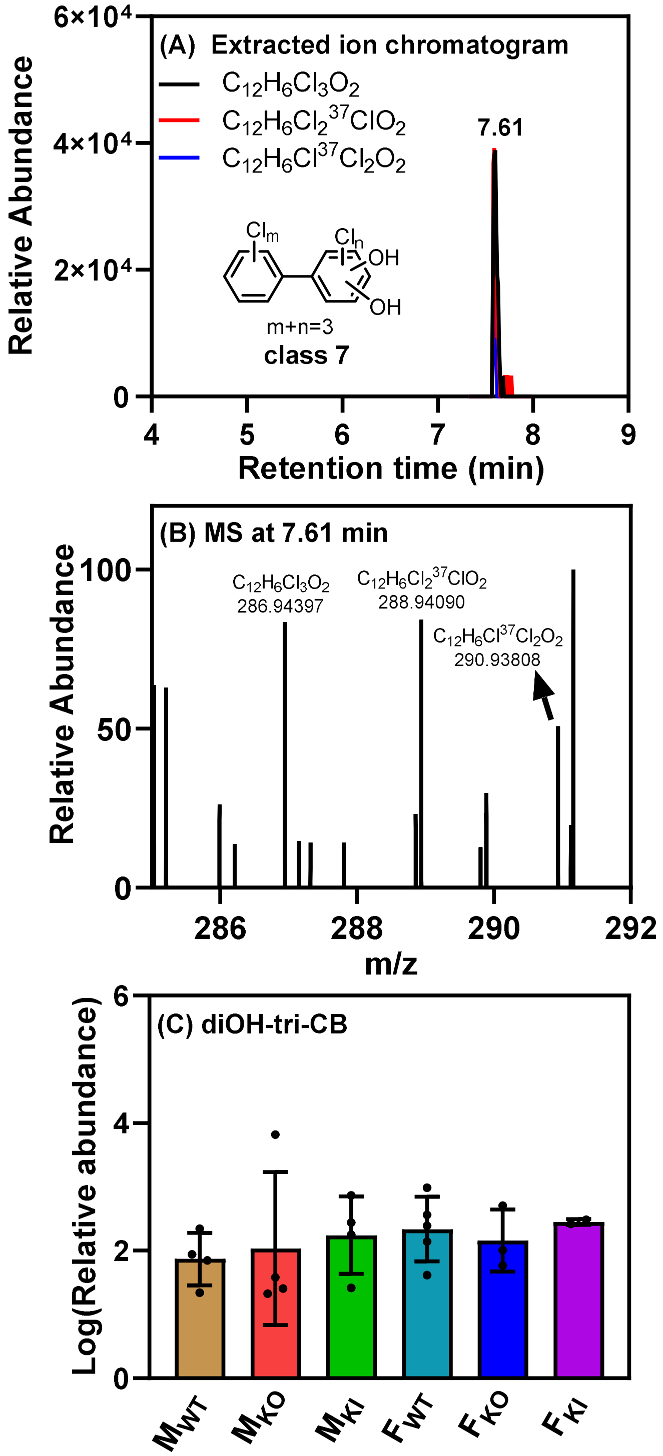


**Figure S7.** A trichlorinated dihydroxylated PCB95 metabolite (diOH-tri-CB; Class 7, **Table 1**) was detected by LC-HRMS subject screening in the intestinal content from male (M) and female (F) wild-type (WT), *Cyp2abfgs*-null (KO), and CYP2A6-transgenic (KI) mice exposed to PCB95. (A) Chromatograms extracted based on the theoretical accurate mass of the top three high-abundance isotope ions of a diOH-tri-CB ($[C_{12}H_6Cl_3O_2]^-$, *m/z* 286.94400 for the monoisotopic ion) show peaks at 7.61 min. The accurate masses of the high-abundance isotope ions at 7.61 min matched the theoretical accurate mass and isotopic pattern of a trichlorinated compound. (C) The relative abundance of the diOH-tri-CB metabolite showed no significant difference between the exposure groups. The LC-HRMS analysis was performed in the negative polarity mode, as described in the Experimental Section.

## References:

(1) Li, X. S.; Bullert, A. J.; Han, W. G.; Yang, W. Z.; Zhang, Q. Y.; Ding, X. X.; Lehmler, H. J. Enantiomeric fractions reveal differences in the atropselective disposition of 2,2',3,5',6-pentachlorobiphenyl (PCB95) in wildtype, cyp2abfgs-null, and CYP2A6-humanized mice. *Chem. Res. Toxicol.* **2023**, *36* (8), 1386-1397.
